# Daily Stress and Heart Rate Variability Among Mindfulness Meditation Practitioners: mHealth Observational Study

**DOI:** 10.2196/78244

**Published:** 2026-05-29

**Authors:** Jo Takezawa, Shixian Geng, Masahiro Fujino, Mika Miyake, Kazutoshi Sasahara, Koji Yatani, Atsushi Niida

**Affiliations:** 1 Interactive Intelligent Systems Laboratory The University of Tokyo Tokyo Japan; 2 Department of Innovation Science School of Environment and Society Institute of Science Tokyo Tokyo Japan; 3 Human Information Science Laboratory NTT (Nippon Telegraph and Telephone Corporation) Communication Science Laboratories Atsugi, Kanagawa Japan; 4 Garmin Health Garmin Japan Ltd. Yokohama, Kanagawa Japan; 5 Laboratory of Molecular Medicine, Human Genome Center The Institute of Medical Science The University of Tokyo Tokyo Japan

**Keywords:** mindfulness meditation, stress reduction, heart rate variability, experience sampling method, smartwatch

## Abstract

**Background:**

Mindfulness meditation has been reported to reduce stress and enhance well-being. However, its effects on heart rate variability (HRV)—a physiological marker of stress—remain underexplored.

**Objective:**

This study aimed to examine how meditation practice is associated with subjective stress, HRV, and their interaction, using mobile health technologies.

**Methods:**

This 3-week observational study included 90 participants—19 meditation practitioners (meditation group), 32 recreational runners as an active control group characterized by lower stress and higher HRV (running group), and 39 individuals without regular meditation or exercise habits (control group). HRV was continuously recorded using Garmin smartwatches. Subjective stress levels and activity states were assessed 3 times daily through a smartphone-based experience sampling method, yielding a total of 4557 responses (mean 50.6, SD 22.8 per participant). From the meditation group, start and end times of 632 daily meditation sessions (mean 33.3, SD 18.3 per participant) were also collected via the app. Standardized questionnaires on stress and related measures were administered at the end of the study period.

**Results:**

The questionnaire survey confirmed that stress levels were significantly lower in both the meditation and running groups compared with controls (median Perceived Stress Scale scores: meditation 21, IQR 17-24; running 22, IQR 19-25; control 25, IQR 21-30; Kruskal-Wallis *P*=.02; adjusted Wilcoxon *P*=.05 and .04, respectively). Smartwatch-derived HRV (root-mean-square of successive differences [RMSSD]) was elevated in the running group relative to controls (median 47.0, IQR 44.0-54.2 and median 42.0, IQR 34.2-47.8, respectively; *P*<.001), whereas no significant difference was observed between the meditation and control groups (median 40.8, IQR 35.5-44.4 and median 42.0, IQR 34.2-47.8, respectively). Bayesian analysis of the experience sampling method data indicated that higher subjective stress levels were associated with a concurrent RMSSD reduction of –2.24 (95% credible interval –3.97 to –0.26) milliseconds. Although this pattern was consistent across groups, the steeper decline of –3.94 (95% CI –7.04 to –0.74) milliseconds was observed only in the running group, likely reflecting their elevated baseline HRV. Additionally, Bayesian modeling of 632 logged meditation sessions revealed an acute RMSSD increase of +4.68 (95% CI 2.96 to 6.38) milliseconds during meditation, with effects maintained for at least 30 minutes post practice.

**Conclusions:**

Although HRV among meditation practitioners did not appear elevated in overall daily life, the ability to increase HRV at arbitrary timings, along with the prolonged residual effect, may correlate with stress reduction. This hypothesis requires further exploration with adequate controls. Despite the preliminary nature of this study due to its limited sample size, our findings highlight the potential of mobile health–based methodologies to capture stress and HRV dynamics in real-world settings.

## Introduction

Mindfulness refers to a psychological state characterized by present-moment awareness and nonjudgmental acceptance of one’s experiences [1]. Although originally rooted in Buddhist traditions, mindfulness meditation (hereafter referred to simply as meditation) has been redefined as a secular mental practice aimed at cultivating mindfulness, and is now widely adopted in nonreligious contexts for its potential to reduce stress and enhance well-being [2,3]. Numerous studies have demonstrated the effectiveness of meditation in reducing perceived stress through self-report measures [4-6]. However, these studies have primarily assessed stress levels in constrained laboratory settings or before and after fixed intervention periods, leaving the impact of daily meditation practice on real-world stress largely unexplored.

Psychological stress is commonly conceptualized as a transactional process in which environmental demands are appraised as taxing or exceeding one’s coping resources, thereby eliciting emotional and physiological responses [7]. In this study, we focus on perceived stress (ie, subjective appraisal) as captured by self-report measures [8]. In addition to retrospective questionnaires, subjective stress levels can be assessed using the experience sampling method (ESM), which is also referred to as ecological momentary assessment [9,10]. While questionnaires are suitable for capturing general trends in perceived stress, ESM enables participants to report their thoughts, emotions, and behaviors multiple times per day in real time, within their natural environments. This approach enables the simultaneous collection of contextual information, facilitating the examination of temporal relationships between stress and other variables in everyday life. Recently, ESM has been used in meditation research to evaluate the stress reduction effects in real-world contexts [11,12]. For example, a recent study used ESM to examine the effects of a brief mindfulness-based intervention and demonstrated that, although no acute changes were observed during the intervention phase, stress decreased at postintervention and follow-up [13].

Heart rate variability (HRV) is widely used as a physiological marker of stress, reflecting autonomic nervous system balance through fluctuations in beat-to-beat intervals (BBIs). Higher stress levels are generally associated with reduced HRV [14,15]. Although HRV has traditionally been measured using electrocardiography (ECG), recent advances in mobile health (mHealth) technologies have enabled widespread use of consumer-grade wearable devices that rely on photoplethysmography (PPG) [16,17]. PPG-based measurements are more susceptible to noise than ECG, particularly under conditions involving motion. Studies comparing wearable-derived PPG measures with ECG in daily-life settings show that agreement is condition-dependent—often higher during low-motion periods, such as sleep—and that PPG-derived indices are not always interchangeable with ECG-derived HRV, underscoring the need for appropriate quality control and cautious interpretation [18,19]. Nevertheless, PPG-based wearables offer practical advantages, including portability, long battery life, and feasibility for long-term monitoring in real-world environments. Their low cost and widespread adoption further enable large-scale population studies, and recent evidence indicates that PPG-derived HRV metrics are sufficiently reliable to capture population-level patterns of autonomic function across age, sex, and circadian rhythms, supporting their use in stress-related research [20].

Laboratory-based studies have consistently demonstrated an inverse association between HRV and perceived stress, particularly under experimentally induced stress conditions. A recent laboratory investigation using PPG-derived HRV from wearable devices has replicated these findings, demonstrating reliable reductions in HRV during controlled cognitive stress tasks and strong concordance with ECG-based measures as well as validated psychological stress scales [21]. However, findings in everyday life settings have been inconsistent [22-25]. More recently, a study integrating ESM with PPG-based wearable devices for continuous HRV monitoring has begun to clarify this relationship in real-world contexts; although effect sizes are generally modest, elevated perceived stress has been shown to be significantly associated with concurrent reductions in HRV [26]. A recent ambulatory study in a clinical population also demonstrated that HRV-based stress detection can meaningfully capture real-world stress states, particularly when individual characteristics, such as age and BMI, are taken into account, supporting the practical utility of wearable HRV for stress assessment beyond laboratory settings [27].

Although transient increases in HRV during meditation have been consistently reported in laboratory settings [28,29], whether long-term meditation practice yields maintained enhancements in HRV remains uncertain, as findings from intervention studies have been mixed. Indeed, an ambulatory randomized controlled trial found no significant effects of mindfulness-based intervention on daily-life HRV indices [30], and a recent meta-analysis reported only small-to-moderate and highly heterogeneous effects on vagally mediated HRV [31]. Consistent with this uncertainty, a recent systematic review of brief mindfulness interventions concluded that evidence for long-term HRV enhancement remains limited and of low quality [32]. Moreover, recent repeated-measures data show that meditation-related HRV modulation emerges transiently during practice, is stronger among experienced practitioners, and does not manifest as elevated baseline or daily-life HRV, indicating predominantly state-dependent and experience-dependent rather than trait-level effects [33]. Notably, most of these insights are derived from laboratory-based measurements or structured intervention designs, and there has been little to no observational research systematically examining HRV patterns in daily-life settings or during routine meditation practice among experienced meditation practitioners. In contrast, there is clear evidence from another behavioral domain—regular physical activity—that long-term, habitual engagement can be reflected in elevated everyday HRV. Physical activity is well established to confer stress-reducing benefits [34,35], and athletes consistently exhibit higher resting and daily-life HRV compared with nonathletic individuals [36,37], providing a useful reference for understanding how continuous practices may shape autonomic regulation in naturalistic settings.

In summary, despite growing evidence linking meditation to stress reduction and HRV elevation, the lack of real-world monitoring has limited our understanding of how these relationships unfold in daily life. We therefore conducted an observational study to investigate the relationship between stress and HRV in experienced meditation practitioners, integrating smartwatch-based HRV monitoring and ESM. The study was conducted on 90 participants consisting of 3 groups—19 meditation practitioners (meditation group), 32 recreational runners as an active control group characterized by lower stress and higher HRV (running group), and 39 individuals with neither a regular meditation nor exercise routine as a passive control group (control group). Over a period of 3 weeks, participants continuously wore Garmin smartwatches to monitor HRV. In parallel, smartphone-based ESM was used to assess momentary perceived stress and activity states throughout daily life. At the end of the monitoring period, participants completed a web-based questionnaire evaluating subjective stress and related factors. Furthermore, for the meditation group, the timing of each daily meditation session was logged via the smartphone app, allowing the capture of HRV dynamics during and after practice. These multimodal datasets were integrated to address the following key questions:

1. Do meditation practitioners exhibit lower levels of perceived stress in daily life, consistent with previous findings?

2. Are HRV levels in daily life elevated among meditation practitioners compared with control participants?

3. Do real-time associations of stress with activity states and HRV differ between meditation practitioners and control participants?

4. During regular meditation sessions, do meditation practitioners show increases in HRV, as reported by laboratory-based studies, and if so, how long are such increases maintained after meditation sessions?

**Figure 1:**
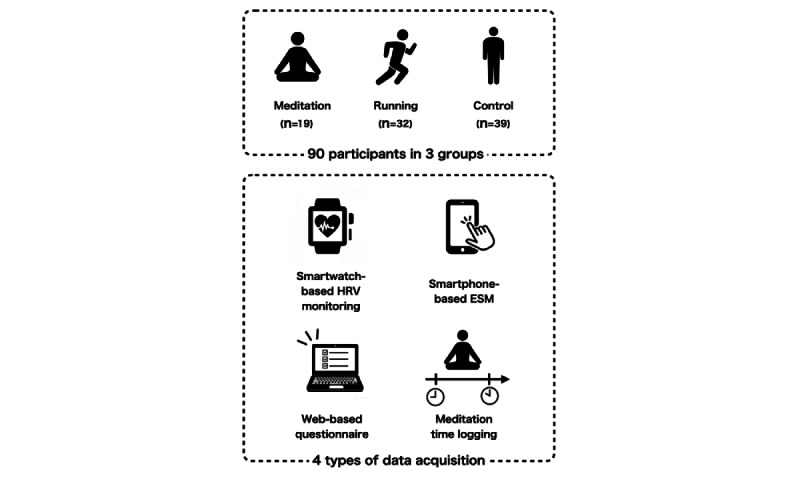
Graphical Summary of our study design. Data were obtained from 90 participants divided into 3 groups—19 meditation practitioners (meditation group), 32 recreational runners (running group), and 39 individuals with neither regular meditation nor exercise routines (control group). Over a 3-week period, participants continuously wore Garmin smartwatches to monitor heart rate variability. In parallel, a smartphone-based ESM was used to assess momentary stress and activity in daily life. At the end of the study, participants completed a web-based questionnaire on subjective stress and related psychological variables. For the meditation group, daily practice times were logged via a smartphone app to enable fine-grained analysis of heart rate variability dynamics during and after meditation. ESM: experience sampling method; HRV: heart rate variability.

Taken together, this study seeks to quantitatively assess how meditation practice is associated with subjective stress, HRV, and their interaction in real-world settings, leveraging mHealth technologies to extend beyond the constraints of controlled laboratory environments (Figure 1).

## Methods

### Participants and Recruitment

We recruited healthy adults aged 18 years and older who did not receive medical treatment or take regular medication. We grouped participants based on the following criteria:

1. Meditation group: Participants must have at least 1 year of experience in secular mindfulness meditation (eg, mindfulness-based stress reduction and mindfulness-based cognitive therapy) or meditation based on traditional schools of Buddhism (eg, Theravada and Zen). They must practice meditation for at least 20 minutes a day, 5 days a week, and meet at least one of the following criteria:

a. Participation in an intensive meditation retreat of at least 1 week.

b. Completion of a teacher training course in the Method of Clinical Meditation [38].

c. Completion of an 8-week mindfulness program (eg, mindfulness-based stress reduction and mindfulness-based cognitive therapy).

2. Running group: Participants must have no regular practice of meditation or yoga, no participation in meditation courses (retreats or multiweek programs), and must run for at least 30 minutes a day, 4 days a week, for more than a year.

3. Control group: Participants must have no regular practice of meditation or yoga, no participation in meditation courses, and exercise no more than 2 days a week for a maximum of 30 minutes per day.

All participants were required to own an iPhone (Apple Inc), as data collection and experience sampling were conducted using UTracker, an in-house iOS app. The app is compatible with Garmin watch models Vivosmart 4, ForeAthlete 245/245M, and ForeAthlete 745. We provided Vivosmart 4 to participants who did not own compatible Garmin watches at the time of the data collection, while those who already owned one of the supported models used their own devices.

We prepared a project website describing the recruitment criteria and study conditions, along with a link to the application page. On the application page, participants provided information regarding their health profiles and gave informed consent electronically before enrollment. Participant recruitment was conducted through multiple channels. Press releases announcing the launch of the project were issued, which also included a call for research participants together with a link to the project description page. The same recruitment information was also disseminated via social media platforms, including Facebook. In addition, recruitment announcements were distributed through a Garmin-operated mailing list targeting Garmin users, which proved particularly effective for enrolling participants in the running group. In addition to these standard recruitment approaches, participants in the meditation group were also recruited through direct contact via the authors’ personal networks.

During a 2-month recruitment period from August 2 to September 30, 2022, a total of 301 individuals applied to participate in the study. To ensure a comparable health profile across the 3 groups, additional selection criteria were applied to the applicants:

- No ongoing medical treatment or regular medication use.
- Age between 25 and 65 years.
- Smoking fewer than 20 cigarettes per day.
- Consuming alcohol fewer than 4 times per week and less than 100 grams per drink.

This selection process resulted in the enrollment of 140 participants (meditation: n=23, running: n=57, and control: n=60) at the start of data collection.

### Data Collection

We collected 3 types of data from 3 participant groups: physiological data from smartwatches, self-reported stress data assessed via ESM, and questionnaire data. For participants in the meditation group, we additionally recorded the start and end times of each meditation session.

We collected physiological and ESM data using our UTracker app, which leverages the Garmin Health SDK to capture BBIs and step counts from Garmin smartwatches, transmitting data to a server. Participants used this app to log data for any 3 weeks between October 12, 2022, and December 21, 2022. The app invoked ESM notifications randomly 3 times within their awake time, prompting participants to report their daily activity states (eating, work, housework, leisure, travel, rest, or other) and subjective stress on 4 levels (no, low, middle, or high stress). Responses submitted within 15 minutes were considered valid. Meditation group participants also recorded meditation session timings via an in-app feature. After the 3-week period, participants completed web-based questionnaires, which were administered via LimeSurvey (LimeSurvey GmbH).

Of the recruited participants, 90 individuals (meditation: 19, running: 32, and control: 39) provided valid smartwatch and ESM data and completed the questionnaires. We therefore used the data from these 90 participants in the subsequent data analysis. The recruitment process, device usage, and demographic characteristics, including group-wise distributions of sex, age, and BMI, are summarized in the supplementary visualization (Multimedia Appendix 1).

### Questionnaire Measures

Physical activity was assessed using the International Physical Activity Questionnaire (IPAQ) short form (7 items) [39]. In accordance with the IPAQ scoring protocol, responses were converted to total metabolic equivalent task minutes per week, with higher values indicating greater physical activity. Sleep quality was assessed using the Pittsburgh Sleep Quality Index (PSQI; 19 items) [40], yielding a global score ranging from 0 to 21, where higher scores indicate poorer sleep quality. Dispositional mindfulness was assessed using the Mindful Attention Awareness Scale (MAAS) [41]. The MAAS consists of 15 items rated on a 6-point Likert scale ranging from 1 (almost always) to 6 (almost never). To align with the study’s analysis strategy, scores were calculated as the total sum of the 15 items (possible range: 15-90), with higher scores indicating higher levels of dispositional mindfulness. Perceived stress was assessed using the 10-item Perceived Stress Scale (PSS) [42], with total scores ranging from 0 to 40, where higher scores indicate greater perceived stress. Well-being was assessed using the World Health Organization Well-Being Index (WHO-5; 5 items) [43], with total scores ranging from 0 to 25, where higher scores indicate greater well-being. All questionnaires were administered in Japanese using validated Japanese versions where available [39-43].

### Calculation of HRV Indexes

From smartwatch data, we derived time-domain indices of HRV as objective markers of stress levels. These HRV indices were used to compare HRV among the 3 groups and to evaluate the association of HRV with ESM-derived stress levels and meditation (hereafter, referred to as the comparative HRV analysis, the ESM-HRV analysis, and the meditation-HRV analysis, respectively).

To calculate the HRV indices, we used the BBI time series provided via the Garmin Health SDK, which is derived from wrist PPG. The “Enhanced BBI” includes a per-beat binary confidence indicator; beats can be flagged as low confidence mainly due to motion artifacts or poor signal quality, and no beat is provided during periods of very high motion [44]. To ensure data reliability, we applied additional rule-based quality filters. We segmented heart rate (HR) interval data into nonoverlapping 5-minute windows starting at 00:00, and retained only those with a beat count between 150 and 1000 and a standard deviation of normal-to-normal interval (SDNN) between 10 milliseconds and 200 milliseconds. We also excluded windows for which the UTracker app returned no step-count values (missing activity flag), as these could not be classified into physical activity states.

Because continuous monitoring occasionally exceeded the prespecified observation period, data length was harmonized by selecting, for each participant, a contiguous 21-day window (3 weeks) that maximized the number of available 5-minute windows. In the comparative HRV analysis, the nonoverlapping 5-minute windows starting at 00:00 were used. For ESM-HRV and meditation-HRV analyses, we prepared different 5-minute windows contained by the 21-day measurement period: the ESM-HRV analysis used 5-minute windows centered on ESM reporting times, while the meditation-HRV analysis used 5-minute windows aligned with the reported start or end times of meditation sessions.

Across all participants, a total of 409,608 5-minute windows containing “Enhanced BBI” data were obtained via the Garmin Health SDK (mean 4551, SD 1927 per participant). Among these, 5242 windows (mean 58, SD 47 per participant) were excluded according to the 3 predefined exclusion criteria: beat count, SDNN, and assignment of a physical activity state. The remaining 322,220 windows (mean 3580, SD 1148 per participant) that fell within the selected 21-day measurement periods were used for the subsequent analyses (Multimedia Appendix 2).

Throughout all the 3 analyses, we primarily focused on the root-mean-square of successive differences (RMSSD), which is known to strongly correlate with high-frequency (HF) HRV and to be sensitive to changes in parasympathetic activity [14]. RMSSD was computed within each 5-minute window by calculating successive differences between adjacent intervals, squaring these differences, averaging the square values, and taking the square root of the mean.

For the comparative HRV analysis, we evaluated the distribution of mean RMSSD across the 3 groups. Mean RMSSD was calculated by averaging RMSSD values from all valid windows during the measurement period for each participant. To ensure the robustness of our findings, additional short-term HRV metrics, including SDNN, percentage of adjacent NN intervals greater than 50 milliseconds, and mean of normal-to-normal intervals (meanNN), were similarly assessed. Long-term HRV was also evaluated using the SDNN, calculated by averaging BBIs within 5-minute windows over a 24-hour period and determining the SD of these means. SDNN provided a stable measure of long-term autonomic activity, complementing short-term metrics by mitigating the influence of transient fluctuations and artifacts [45].

To further investigate HRV differences while accounting for variations in physical activity levels across the groups, we stratified RMSSD by physical activity states. Using smartwatch-derived step counts and self-reported bedtime and wake-up time collected via the UTracker app, we categorized 5-minute windows into 4 states: run (≥400 steps), walk (50-399 steps), rest (10-49 steps or <10 steps outside sleep), and sleep (<10 steps during sleep). Windows lacking step information could not be assigned a physical activity state and were therefore excluded during the data-cleaning process. From windows assigned to each state, we calculated the mean RMSSD for each participant and compared the distributions among the 3 groups.

In addition to the HRV indices, we calculated HR for each physical activity state (sleep, rest, walk, and run) from the meanNN (in milliseconds) by converting it to beats per minute (HR=60,000/meanNN). For each participant, state-specific HR values were summarized as the median across all eligible 5-minute windows within each state over the 21-day measurement period. Resting HR was operationalized as the median HR during the rest state.

In the meditation-HRV analysis, we examined alterations in autonomic nervous system activity using frequency-domain metrics. Low-frequency (LF; 0.04-0.15 Hz) and HF (0.15-0.40 Hz) power were calculated using Welch periodogram after cubic spline resampling at 4 Hz for each 5-minute window. However, frequency-domain metrics derived from wrist PPG are subject to greater measurement uncertainty than those obtained from ECG [46,47]. Therefore, these metrics are reported as supplementary descriptive indices rather than primary outcomes.

### Statistical Analysis

Normality was first assessed using the Shapiro-Wilk test. Because several variables deviated from normality and included outliers despite preprocessing, nonparametric tests were used to compare questionnaire scores and HRV indices among the 3 groups. To ensure comparability across outcomes, the same nonparametric approach was applied to all outcomes. Shapiro-Wilk *P* values, as well as the numbers of missing observations and IQR-based outliers, are provided in Multimedia Appendix 3. For group comparisons, Kruskal-Wallis tests were used for overall differences, followed by pairwise Wilcoxon rank-sum tests with multiple-comparison adjustment using the Shaffer procedure [48]. Effect sizes were calculated as epsilon-square (*ε*^2^) for Kruskal-Wallis tests, and rank-biserial correlation (*r*) together with the common-language effect size for pairwise Wilcoxon tests. As a sensitivity analysis, we additionally conducted parametric tests (Welch ANOVA followed by Games-Howell post hoc tests), which yielded results consistent with those from the nonparametric analyses.

To account for the effect of age on HRV, we also conducted an analysis of covariance (ANCOVA) with age included as a covariate (age entered as a continuous variable; group as the main factor). Effect sizes were quantified as partial eta-square (*η*^2^), and a group-by-age interaction term was examined to assess the assumption of homogeneous regression slopes. In addition, given the unequal group sizes and the potential presence of heteroskedasticity, we performed a sensitivity analysis using ANCOVA with HC3 heteroskedasticity-consistent SEs.

For analyses of ESM-reported stress levels, stress ratings were dichotomized (no-low vs middle-high). Furthermore, 2-sided binomial tests were conducted at the pooled and group-specific levels for each activity state to examine whether the observed proportion of middle-high stress differed from the expected proportion, using the corresponding pooled or group-specific proportion as the binomial parameter. Separately, group-level comparisons of the meditation and running groups against the control group were performed using binomial tests with the control group proportion as the reference parameter. *P* values from all binomial tests were adjusted for multiple comparisons using the Holm procedure. Effect sizes were quantified using Cohen *h*.

The *P* values and effect sizes for all statistical tests are provided in Multimedia Appendices 3-11. All statistical analyses were performed using Python (version 3.12) with the *SciPy* and *statsmodels* libraries.

### Hierarchical Bayesian Analysis

In the ESM-HRV analysis, we examined differences in HRV across the 2 stress states, no-low and middle-high stress, by using a hierarchical Bayesian model. The model was specifically designed to isolate HRV variations associated with stress states while accounting for interindividual differences in baseline HRV (Multimedia Appendix 12). In the model, *m*, *i*, and *j* index stress states, participants, and observations, respectively. As the observed variable, the model has *mⱼ*, *iⱼ*, and *eⱼ*, which represent the stress state, participant, and RMSSD value in the *j*-th observation. The model assumes that *eⱼ* is sampled from a normal distribution with a mean of *βⱼ* defined as the sum of the mean RMSSD for the observed state (*αⱼ*) and the residual associated with the observed participant (*rⱼ*). By focusing on *αₘ* as a parameter corresponding to the mean RMSSD for state *m*, we estimated the posterior distributions of *αₘ* and the differences in *αₘ* by running Markov Chain Monte Carlo (MCMC).

Additionally, we extended the analysis to examine HRV differences between the 2 stress states within each of the 3 participant groups. For this purpose, the model was adapted such that m indexes 6 states, defined by the combination of the 2 stress levels and the 3 groups. In the meditation-HRV analysis, we examined HRV changes across the following meditation phases: the 10-minute before meditation phase, the during meditation phase, and the after meditation phases with 10-minute intervals up to 60 minutes post meditation. Similarly, the model was adjusted to allow *m* to represent the respective meditation phases.

In the meditation-HRV analysis, we further investigated time-series HRV profiles across meditation sessions of varying durations. For this purpose, we extended the model to assume temporal dependencies among parameters corresponding to mean RMSSD (Multimedia Appendix 13). In the model, *t*, *k*, *i*, and *j* index time points, meditation duration, participants, and observations, respectively. As the observed variable, the model has *tⱼ*, *kⱼ*, *iⱼ*, and *eⱼ*, which represent the time points, meditation duration, participant, and RMSSD value in the *j*-th observation. The parameter corresponding to the mean RMSSD for the time point *t* and the meditation length *k*, *αₜᵏ*, is sampled from a normal distribution with a mean of the parameter of the previous time point, *αₜ₋₁ᵏ*, though the parameter of the initial time point, *α₁ᵏ*, depends on a hyper-parameter *α*. Taking the start or end of meditation as time 0, RMSSD was computed using start or end time-aligned windows spanning from –20 to +55 minutes. After grouping meditation sessions by duration in 10-minute intervals, MCMC sampling was conducted to estimate posterior medians and 95% credible intervals of *αₜᵏ*, generating time-series HRV profiles for each duration group.

In all the analyses, MCMC sampling was performed on RStan (version 2.32.6; Stan Development Team), with parameter settings including 4 chains, 1000 burn-in iterations, and 2000 total iterations. MCMC convergence was confirmed using the Gelman-Rubin diagnostic.

### Ethical Considerations

This study was approved by the Ethics Review Board of the Institute of Medical Science, The University of Tokyo (approval 2022-11-0629) and the Human Subject Research Ethics Review Committee of Tokyo Institute of Technology (approval 2022154). Participants provided informed consent by submitting their application after reading the study explanation on the recruitment website. Participation was voluntary, with withdrawal permitted at any time without justification.

As compensation, participants who were provided with Garmin smartwatches retained the devices after the data collection period. Participants who used their own compatible devices received a Garmin electronic coupon.

## Results

### Questionnaire Data Analysis

**Figure 2:**
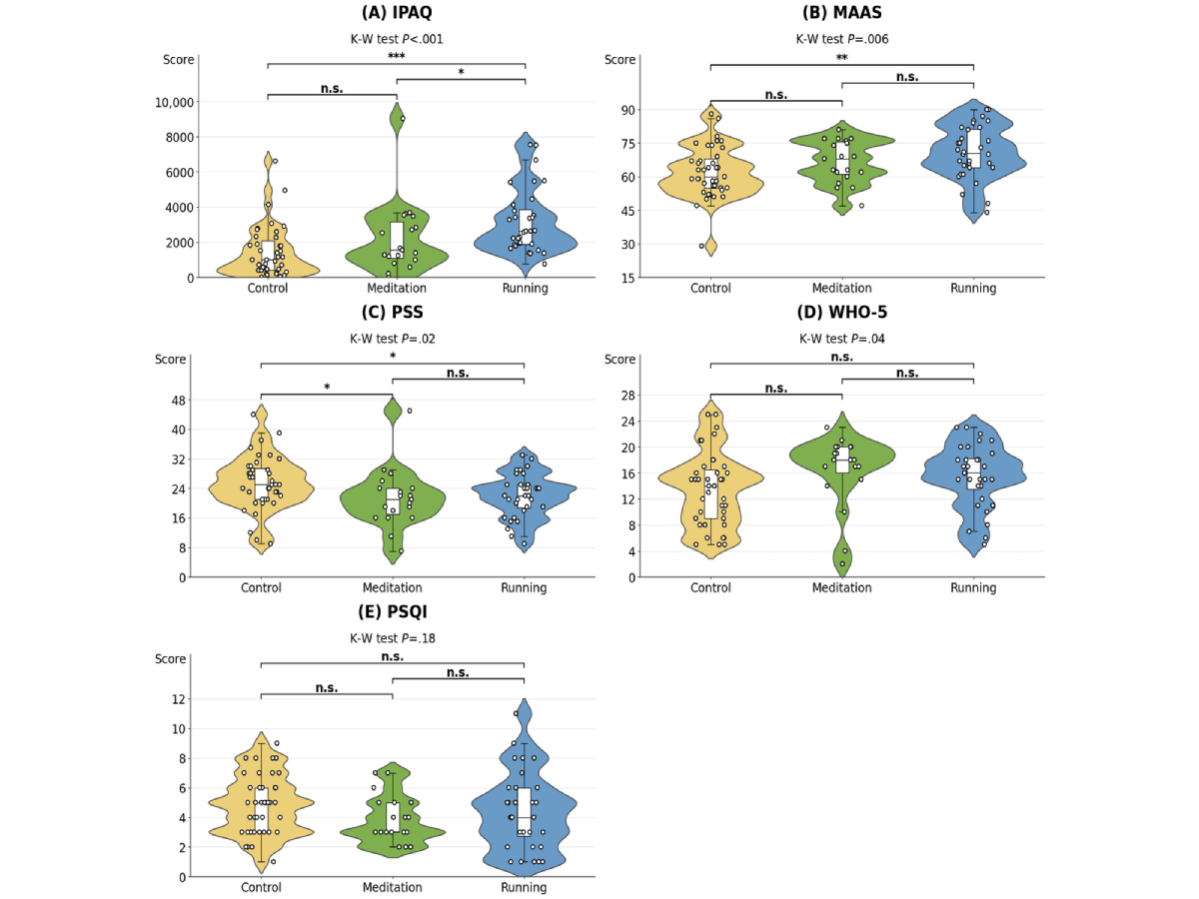
Comparative analysis of the 5 questionnaire scores. The horizontal line within each violin plot denotes the median, while the vertical line or shaded region represents the IQR, encompassing values from the 25th to 75th percentiles. Points represent scores from each participant in the control, meditation, and runner groups. (A) High International Physical Activity Questionnaire scores indicate high physical activity levels, (B) high Mindful Attention Awareness Scale scores indicate high mindfulness levels, (C) low Perceived Stress Scale scores indicate low perceived stress levels, (D) high World Health Organization Well-Being Index scores indicate high psychological well-being levels, and (E) low PSQI scores indicate better sleep quality. Significant differences were assessed using Kruskal-Wallis tests followed by Wilcoxon test. IPAQ: International Physical Activity Questionnaire; K-W: Kruskal-Wallis; MAAS: Mindful Attention Awareness Scale; n.s.: not significant (*P*>.05); PSQI: Pittsburgh Sleep Quality Index; PSS: Perceived Stress Scale; WHO-5: World Health Organization Well-being Index. *P<.05, **P<.01, ***P<.001 in the Wilcoxon test.

We first compare subjective stress levels and related factors across the 3 groups, based on scores from the 5 questionnaires (Figure 2; refer to Multimedia Appendices 4 and 5 for nonparametric analyses). The IPAQ results confirmed that the running group demonstrated significantly higher physical activity than the control group (median 2625, IQR 1885-3871 and median 990, IQR 424-2075, respectively; Kruskal-Wallis test *P*<.001; Wilcoxon test with Shaffer correction *P*<.001), with the meditation group scoring between the two (median 1548, IQR 1089-3150). In the MAAS questionnaire, the running group had significantly higher mindfulness scores than the control group (median 70.5, IQR 64-81 and median 60, IQR 55-68, respectively; Kruskal-Wallis *P*=.006; Wilcoxon *P*=.007). The meditation group also showed higher scores, although the difference did not reach the significance cutoff (median 68, IQR 61-76; Wilcoxon *P*=.10).

The PSS results indicate that both the meditation and running groups experienced significantly lower subjective stress compared to the control group (meditation: median 21, IQR 17-24; running: median 22, IQR 19-25; and control: median 25, IQR 21-30; Kruskal-Wallis *P*=.02; Wilcoxon *P*=.05 and .04, respectively). In the WHO-5 questionnaire, a significant difference existed among the 3 groups (mediation: median 18, running: median 16, and control: median 14; Kruskal-Wallis *P*=.04), and both the meditation and running groups showed higher well-being scores than the control group with marginal significance (Wilcoxon *P*=.07 and .08, respectively). We observed no significant patterns in the PSQI questionnaire (mediation: median 3, IQR 3-5; running: median 4, IQR 3-6; control: median 5, IQR 3-6; Kruskal-Wallis *P*=.18).

These findings suggest that both meditation and physical exercise contributed to the reduction of stress, together with enhanced mindfulness and well-being.

### HRV Data Analysis

**Figure 3:**
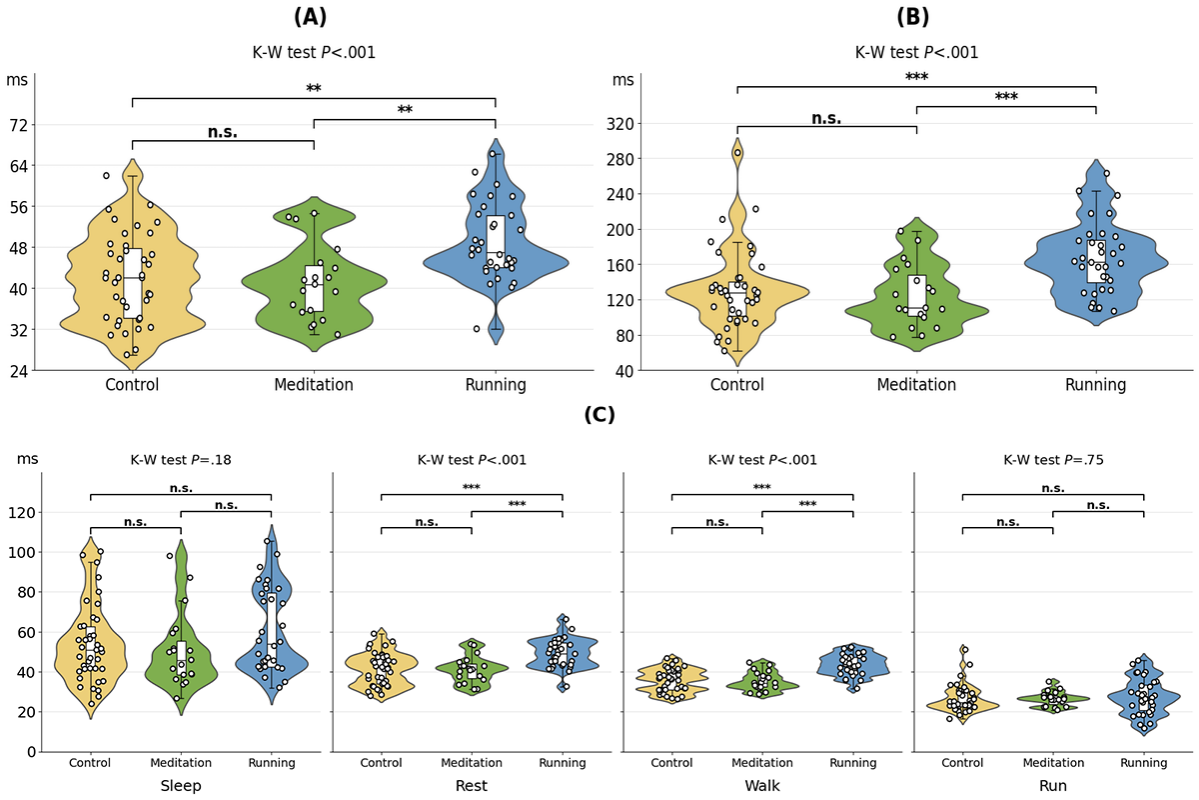
Comparative analysis of the heart rate variability indexes. For each participant, the median values of various heart rate variability indices were calculated from smartwatch-derived beat-to-beat intervals, and their distributions were compared across 3 groups—control, meditation, and runner. The indices include (A) 5-min root-mean-square of successive differences, (B) 24-hour standard deviation of normal-to-normal interval, and (C) root-mean-square of successive differences stratified by 4 physical activity states (sleep, rest, walk, and run), categorized using smartwatch-derived step counts and self-reported sleep times. Figure presentation and statistical tests were conducted in the same manner as in Figure 2. Significant differences were assessed using Kruskal-Wallis tests followed by Wilcoxon test. K-W: Kruskal-Wallis; n.s.: not significant (*P*>.05). *P<.05, **P<.01, ***P<.001 in the Wilcoxon test.

We next compared smartwatch-derived HRV among the 3 groups (Figure 3; refer to Multimedia Appendices 4 and 6 for nonparametric analyses, and Multimedia Appendices 9-11 for parametric analyses). Comparison of median RMSSD showed that the running group exhibited significantly higher RMSSD than the control group (Figure 3A; running: median 47.0, IQR 44.0-54.2, control: median 42.0, IQR 34.2-47.8, respectively; Kruskal-Wallis *P*<.001; Wilcoxon *P*<.001), In contrast, the meditation group exhibited RMSSD levels comparable with those of the control group (mediation: median 40.8, IQR 35.5-44.4, and control: median 42.0, IQR 34.2-47.8). We also found a consistent pattern for other short-term HRV metrics, and SDNN, an HRV metric calculated over 24-hour periods (Figure 3B).

Since HRV is affected by the intensity of physical activity, it is possible that these results merely reflect the fact that the running group engaged in different physical activity intensities. To correct for such effects, we categorized 5-minute windows into 4 activity states—sleep, rest, walk, and run—using smartwatch step counts and self-reported sleep data. We calculated mean RMSSD for each activity state and compared group-level distributions (Figure 3C). The running group consistently displayed higher RMSSD during the rest state (running: median 49.1, IQR 43.7-54.5, control: median 42.0, IQR 34.1-47.2, meditation: median 41.2, IQR 36.8-43.9; Kruskal-Wallis *P*<.001; Wilcoxon *P*<.001) and the walk state (running: median 43.5, IQR 39.7-47.4, control: median 36.5, IQR 31.1-40.6, meditation: median 34.4, IQR 32.4-38.5; Kruskal-Wallis *P*<.001; Wilcoxon *P*<.001), compared with the other groups.

It is well-established that HRV is associated with age [49]. To account for potential age-related effects when examining group differences in HRV, we conducted an ANCOVA with age included as a covariate (Multimedia Appendix 7). The results of the ANCOVA indicated that the observed group differences in HRV remained statistically significant after adjusting for age, confirming the robustness of our findings to age correction. Because RMSSD is known to be associated with HR [50], we also additionally compared HR across the 3 groups within each activity state (Multimedia Appendix 14). HR showed group differences that were consistent with those observed for RMSSD; notably, a significantly lower resting HR was observed exclusively in the running group. These findings indicate that group differences in RMSSD are accompanied by corresponding differences in HR, consistent with known HR-HRV coupling.

Taken together, our results suggest that physical exercise is associated with higher HRV, while meditation practice has no apparent effects on overall HRV patterns in daily life.

### ESM Data Analysis

**Figure 4:**
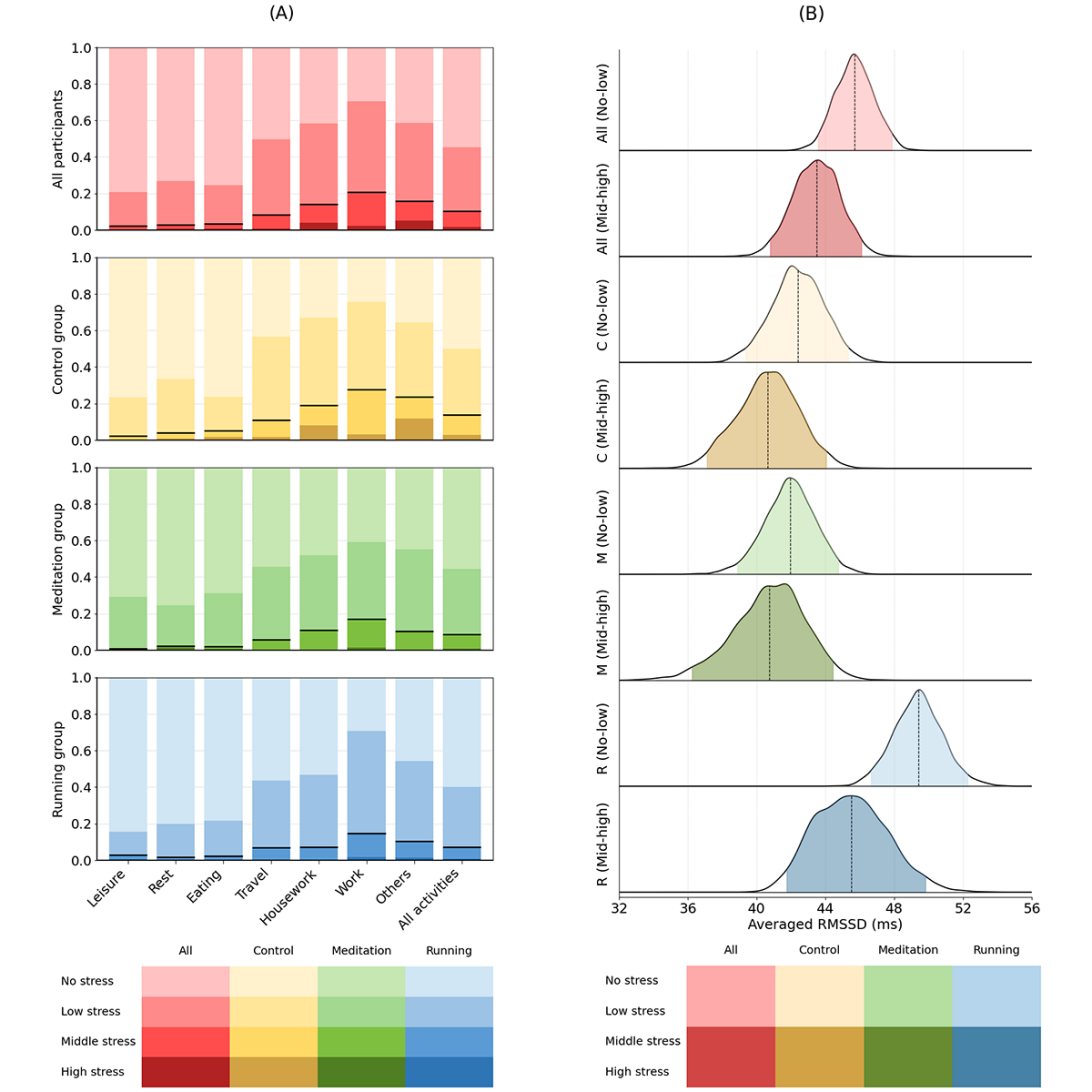
Associations between stress levels, daily activities, and heart rate variability. (A) Stress level distribution by activity type. The proportion of self-reported stress levels across 7 daily activity states, derived from 4557 experience sampling method responses. The vertical axis denotes the proportions of 4-staged stress levels, while the horizontal axis represents activity types. Horizontal black lines across the bars indicate the overall middle-high stress proportion. (B) root-mean-square of successive differences (RMSSD) density plots by stress levels. Posterior density plots of averaged RMSSD under no-low stress and middle-high stress conditions. RMSSD was calculated using 5-minute windows centered on reporting times. A hierarchical Bayesian model (Multimedia Appendix 12) was used to account for variability in response frequency and baseline heart rate variability across participants. Posterior densities of αₘ were estimated using Markov Chain Monte Carlo, with shaded regions denoting 95% credible intervals and vertical dotted lines indicating posterior medians. C: control; M: meditation; R: running.

In this study, we also collected daily activity states and stress levels by ESM to analyze their associations (Figure 4A; Multimedia Appendices 8 and 15). ESM samplings were randomly administered 3 times per day, generating 4557 responses (51.78 per participant on average). Among all the responses, 10.3% (471/4557) reported middle-high stress. Using the overall proportion of middle-high stress responses as a baseline, stress levels were higher during housework (54/386, 14%; adjusted *P*=.05, based on a binomial test) and work (280/1352N, 20.7%; *P*<.001), and lower during leisure (20/864, 2.3%; *P*<.001), rest (21/734, 2.9%; *P*<.001), and eating (16/472, 3.4%; *P*<.001). This pattern was consistent across groups, although the proportion of middle-high stress responses was lower in the running group (121/1680, 7.2%; *P*<.001) and meditation group (76/882, 8.6%; *P*<.001) compared with the control group (274/1995, 13.7%), in line with the questionnaire findings. We also examined the relationship between self-reported stress levels and RMSSD recorded at reporting times (Figure 4B).

To account for interparticipant variations in baseline HRV, we used a hierarchical Bayesian approach to estimate posterior distributions of the average RMSSD for the 2 different stress states—no-low stress and middle-high stress (Multimedia Appendix 12). The analysis revealed that RMSSD was lower during middle-high stress states compared with no-low stress states, confirming the ecological validity of HRV as a stress indicator. We additionally sampled the posterior distribution of the difference between the 2 parameters, whose posterior median estimated the RMSSD difference as −2.242 (95% credible interval −3.967 to −0.260) milliseconds (Multimedia Appendix 16). This trend was observed across all groups, with the running group showing a more pronounced decrease of −3.939 (95% credible interval −7.041 to −0.737) milliseconds, likely due to higher baseline RMSSD values.

Collectively, our ESM analysis demonstrated that subjective stress levels are associated with both daily activity states and HRV.

### Analysis of HRV and Meditation

From the meditation group, we collected information about start and end times of 632 daily meditation sessions (33 per participant on average) using the smartphone app. We investigated HRV changes during meditation by integrating the meditation timing information with the heartbeat data obtained from smartwatches (Multimedia Appendix 17).

**Figure 5:**
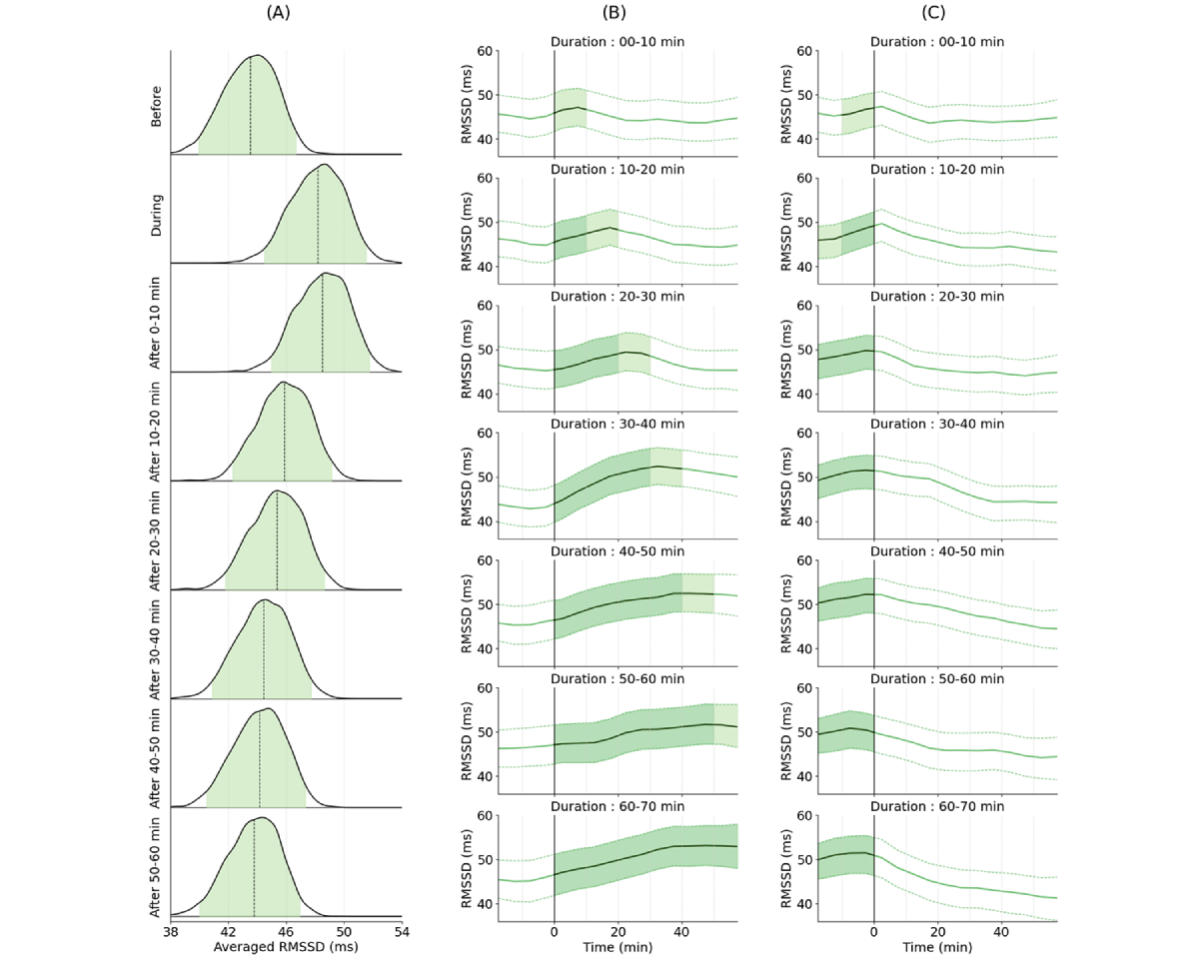
Variation of heart rate variability levels during daily meditation practices. (A) Posterior density plots illustrating the average root-mean-square of successive differences (RMSSD) across different phases of meditation practice. RMSSD data were categorized as follows: the 10-minute before meditation phase, the during meditation phase, and the after meditation phases with 10-minute intervals up to 60 minutes post meditation. RMSSD was computed using start time-aligned 5-minute windows for the before and during phases, while end time-aligned windows were used for the after phases. The data were analyzed and visualized as in Figure 4B. (B) Start-aligned time-series RMSSD profiles across meditation practices of varying durations. Assuming the start of meditation as time 0, RMSSD was calculated using start time-aligned windows spanning from −20 to +55 minutes. Profiles were grouped by meditation duration in 10-minute intervals, and Markov Chain Monte Carlo–based hierarchical Bayesian analysis (Multimedia Appendix 13) was used to estimate posterior medians and 95% credible intervals of αₜᵏ, represented by solid and dotted lines, respectively. Horizontal time scales were aligned with the centers of the RMSSD windows. The shaded regions between the dotted lines denote the during meditation phase, while the lightly shaded areas indicate that the results are based on data partially originating from outside the during meditation phase. (C) End-aligned time-series RMSSD profiles assuming the end of meditation as time 0 and based on end time-aligned windows, visualized in the same manner as in panel (B). RMSSD: root-mean-square of successive differences.

From each participant in the meditation group, RMSSD was computed for the 10-minute period before meditation, the period throughout meditation, and the periods in 10-minute intervals up to 60 minutes after meditation. Similarly to the ESM data analysis, we used hierarchical Bayesian models (Multimedia Appendix 12; also refer to Multimedia Appendix 13 for the time-series extension) to account for interparticipant variations in baseline HRV and calculate posterior distributions of parameters corresponding to averaged RMSSD values for each period (Figure 5A). As a result, RMSSD showed an increase during meditation, with the posterior median estimated at 4.675 (95% credible interval 2.962-6.380; Multimedia Appendix 18). Remarkably, RMSSD required more than 30 minutes to return to baseline levels after meditation, suggesting a prolonged residual effect.

We also examined RMSSD profiles across meditation sessions of varying durations. Meditation sessions were stratified into 10-minute intervals based on their duration, and time-series RMSSD trajectories were computed using a hierarchical Bayesian approach (Figure 5B). The analysis revealed a progressive increase in RMSSD that persisted throughout the meditation period. Upon stopping meditation, RMSSD gradually decreased toward baseline levels, underscoring the persistence of residual effects.

To further characterize the physiological changes during meditation, we analyzed frequency-domain indices, evaluating both their absolute distributions and their baseline-adjusted shifts (Multimedia Appendix 19). During meditation, both HF and LF power showed slight increases, with the elevations most pronounced in the early postmeditation period (after 0-10 min). Thereafter, HF gradually approached baseline levels, whereas LF returned to baseline after 10-20 min and subsequently decreased below baseline. These patterns also suggest transient alterations in autonomic nervous system activity following meditation.

In summary, our analysis revealed that during daily meditation practice, practitioners experience an increase in HRV, which may persist for a few tens of minutes after meditation.

## Discussion

### Principal Findings

In this mHealth-based observational study, we integrated 3 weeks of continuous smartwatch-derived HRV monitoring with smartphone-based experience sampling to examine stress-HRV dynamics in meditation practitioners, recreational runners, and controls. Questionnaire and ESM data consistently indicated lower perceived stress in both the meditation and running groups relative to controls. However, daily-life HRV patterns differed; recreational runners showed higher HRV than controls, whereas meditation practitioners exhibited daily-life HRV levels comparable with controls. Across all groups, higher momentary stress was associated with concurrent reductions in HRV. Finally, within the meditation group, HRV increased during meditation sessions and remained elevated for tens of minutes after practice, suggesting a residual physiological effect detectable in naturalistic settings.

### Interpretation in the Context of Previous Studies

Our questionnaire-based assessments indicated that perceived stress levels were lower in both the meditation and running groups compared with the control group, consistent with previous studies reporting stress-reducing effects of both meditation practice [2,3] and regular physical activity [34,35]. However, reduced subjective stress did not necessarily correspond to higher smartwatch-derived HRV in daily life. In fact, daily-life HRV levels were comparable between meditation practitioners and controls, suggesting that perceived stress and average daily HRV are not simply or linearly related.

Although multiple intervention studies have examined the impact of meditation practice on HRV, their findings have been mixed; some report increases in daily-life HRV among meditation groups relative to controls, while others observe no significant differences [28,31,32]. In our observational study involving experienced meditation practitioners, we found no evidence of a general increase in HRV throughout daily life. This interpretation is consistent with recent repeated-measures evidence showing that meditation primarily modulates HRV at the state level during meditation, particularly among experienced practitioners, even in the absence of elevated baseline HRV [33].

In contrast, and consistent with previous reports [36,37], the running group exhibited higher HRV as well as lower resting HR. This HRV elevation may reflect long-term cardiovascular and autonomic adaptations associated with endurance training, often referred to as an athlete’s heart [51], including increased stroke volume, resting bradycardia, and enhanced vagal tone. Such physiological adaptations are likely to exert a stronger and more persistent influence on daily-life autonomic regulation than psychological training alone, potentially explaining the long-term HRV elevation observed in the running group.

To further explore the relationship between stress and HRV in daily life, we used a smartphone-based ESM integrated with smartwatch-derived HR monitoring. ESM also captured daily activities, allowing us to examine the links between stress and activity states. Across all groups, elevated stress levels were associated with reduced real-time HRV, in line with a previous study [26], as well as with specific stress-related activities such as work.

Consistent with the questionnaires, ESM confirmed that both meditation and running groups experienced lower stress levels in daily life compared with controls. While recreational runners showed more pronounced stress-related reductions in HRV, likely due to elevated baseline levels, meditation practitioners and controls exhibited comparatively smaller shifts.

Previous work has demonstrated that meditation training can attenuate HRV decreases during standardized cognitive stress tasks [52], suggesting that meditation may modulate stress appraisal rather than baseline autonomic function per se. Together, these findings raise the possibility that meditation practitioners experience identical daily-life stressors as less physiologically taxing, resulting in attenuated HRV responses. The absence of clear group differences in overall daily-life HRV does not necessarily contradict this interpretation. In naturalistic settings, stressors of sufficient intensity to elicit marked autonomic responses may occur relatively infrequently, and averaging across heterogeneous daily contexts may obscure group-level differences that emerge primarily under acute stress conditions.

For the meditation group, we also examined changes in HRV during daily meditation practice by integrating HRV data with meditation timing information collected via a smartphone app. We observed a significant increase in HRV during meditation. This finding is broadly consistent with laboratory-based studies reporting acute HRV increases during meditation [28,29,32,33], and extends the previous work by demonstrating such changes in naturalistic, daily-life settings.

Notably, our analysis also demonstrated that the HRV increase persisted for a few tens of minutes afterward, suggesting a substantial residual effect. To our knowledge, such a residual HRV elevation after meditation has not been previously documented. By capturing meditation practices in a naturalistic, daily-life context, this study provides unique insights into the physiological dynamics of meditation. Meditation practitioners’ ability to increase HRV at an arbitrary timing, along with the prolonged residual effect, may correlate with stress reduction; however, this hypothesis warrants further investigation with adequate controls.

In the meditation-HRV analysis, changes were observed not only in the time-domain but also in the frequency-domain HRV indices. The concurrent increases in HF and LF observed during meditation and immediately after meditation are consistent with laboratory studies reporting enhanced parasympathetic activity and elevated overall HRV during meditative states [53]. HF gradually returned toward baseline after meditation, suggesting recovery from this parasympathetic-dominant state, while the delayed decrease in LF below baseline may reflect a transient reorganization of autonomic regulation after meditation ended. However, these interpretations should be made with caution. Frequency-domain indices derived from wrist-worn PPG may be less precise than those obtained from ECG [54,55], and the physiological interpretation of frequency-domain indices as markers of autonomic activity remains debated [14,56]. Therefore, although these observations may also support the residual effect of meditation, we refrain from drawing strong conclusions regarding the underlying mechanisms.

In addition, the observed HRV increase may be partly attributable to respiration-related heart rate variability (RespHRV) induced by changes in breathing pace [57,58]. Under typical conditions, RespHRV is primarily reflected in the HF band; however, slow and regular breathing, which commonly occurs during meditation, can shift respiratory oscillations toward lower frequencies and thereby contribute to increases in LF power. Because respiratory data were not collected in this study, we were unable to directly assess the contribution of RespHRV to the observed HRV changes. Recent studies have demonstrated that emerging wearable technologies, such as chest-mounted or textile-based respiratory sensors, enable direct and continuous measurement of breathing patterns in daily-life settings [59]. Incorporating such respiratory sensing technologies in future studies will help disentangle respiration-driven HRV modulation from other autonomic mechanisms and further clarify the physiological basis of meditation-related HRV changes.

From a methodological perspective, this study highlights the utility of Bayesian analysis for wearable PPG-derived HRV data. While most HRV studies have relied on ECG-based devices, this study used a Garmin smartwatch with PPG sensors, enabling the collection of 3 weeks of continuous data. Although the reliability of wearable PPG-derived HRV metrics has been debated due to susceptibility to noise [16-19], the combination of extensive data collection and robust statistical approaches allowed us to extract meaningful physiological insights. In particular, the hierarchical Bayesian approach used in our ESM-HRV and meditation-HRV analyses was well-suited to this setting, as it explicitly accounts for the nested and repeated-measures structure of the data. By partially pooling information across individuals, the model reduces the influence of noisy or uncertain observations at the individual level while preserving between-participant heterogeneity in baseline HRV. This framework enables more stable estimation of covariate-associated effects under realistic measurement uncertainty, which is especially relevant for wearable PPG-derived signals collected in daily life. Aligned with a recently published work [60], our achievement highlights the potential of Bayesian approaches to enhance the utility of wearable PPG-based devices in HRV research.

### Limitations

Despite these promising results, our study remains preliminary, with several limitations requiring attention. First, unequal sample sizes across the 3 groups may have introduced imbalances in statistical power and estimation precision, which should be considered when interpreting between-group comparisons. Moreover, our recruitment procedures may have introduced potential biases in the composition of participant groups. A substantial proportion of the running group used their own Garmin smartwatches for data collection, while the control and meditation groups primarily used study-provided devices (Multimedia Appendix 1). This discrepancy arose from recruiting runners via Garmin users’ mailing list. This could influence the results due to differences in device models and the users’ familiarity with the devices, although we confirmed that recreational runners wearing different device models showed no significant difference in HRV levels (data not shown).

The meditation group also harbored potential recruitment biases. Participants in the meditation group were recruited through calls on social media and direct outreach to potential candidates. In the recruitment process, we noticed that more experienced meditation practitioners were often reluctant to wear a smartwatch on a daily basis, which may have biased our sample toward individuals with less meditation experience. Additionally, the meditation group included participants practicing different meditation styles, which may have introduced heterogeneity. In the questionnaire analysis, the mindfulness scores showed no significant difference, which may reflect not only the small sample size but also these concerns related to participant recruitment.

It is also important to consider that the 3 groups may have differed in covariates that could have influenced our analyses (Multimedia Appendix 1). Although age-adjusted sensitivity analyses using ANCOVA yielded results consistent with the primary nonparametric analyses, future studies should recruit larger and more balanced samples and incorporate more comprehensive covariate adjustment to strengthen the robustness and interpretability of the findings.

Finally, because respiration was not directly measured, we cannot fully disentangle stress-related autonomic changes from respiration-driven HRV modulation, particularly during meditation sessions. Moreover, the frequency-domain metrics reported in this study (HF and LF) are derived from PPG and are subject to greater estimation error than ECG-based measures, particularly under free-living conditions. Therefore, the interpretation of these indices should ideally be supported by validation against ECG-derived HRV measurements together with concurrent respiratory monitoring.

### Conclusions

In this study, we used an mHealth framework integrating smartwatch-based physiological monitoring with smartphone-based experience sampling to examine how meditation practice is associated with subjective stress, HRV, and their interaction in real-world settings. We showed that meditation practitioners reported lower perceived stress but did not exhibit higher daily-life HRV than controls. Real-time associations between stress, activity states, and HRV were also comparable between the meditation and control groups. HRV analysis integrated with meditation timing logs showed that daily meditation practice was accompanied by transient increases in HRV, consistent with laboratory-based studies. Notably, we extend previous work by demonstrating that the HRV elevation persists for several tens of minutes after meditation practice.

Building on these findings, this study illustrates that mHealth-based approaches can capture dynamic relationships among subjective stress, daily activities, and autonomic physiology outside laboratory environments. Continuous wearable sensing yielded dense physiological time series, and the integration of these data with in situ self-reports enabled the examination of stress-HRV associations at fine temporal resolution. Such multimodal data allowed us to characterize both baseline daily-life patterns and short-term physiological changes associated with meditation practice under naturalistic conditions.

From a digital health perspective, quantifying acute and short-lived physiological changes surrounding meditation sessions provides a basis for evaluating the timing and immediate after-effects of daily practice. The findings of this study suggest that mobile systems combining wearable sensors and self-reports may enable individual-level assessment of stress-HRV coupling and short-term recovery signals following meditation. Future studies incorporating additional physiological signals, such as respiration, and intervention-based designs will be necessary to further elucidate underlying mechanisms and to assess the practical utility of these integrated mHealth approaches for stress management.

## Appendix

Multimedia Appendix 1 Characteristics of the recruited participants. (A) Recruitment flow chart of participants assigned to three groups: meditation (M), running (R), and control (C). (B) Sankey diagram illustrating the relationships between Garmin usage, group classification, and sex distribution. GU represents Garmin users, who participated in the study with their own smartwatches, while NGU represents non-Garmin users, who participated using provided smartwatches. The width of the flows corresponds to the number of participants transitioning between categories, with numbers in parentheses indicating the total participants in each category. (C) Age and (D) Body Mass Index (BMI) distribution across the three groups and differences in Garmin usage. In the violin plots (as presented in Figure 2), white dots represent female participants, while black dots represent male participants. BMI was calculated as weight (kg) divided by the square of height (m²).

Multimedia Appendix 2 (A) Stacked bar chart showing the distribution of all 5-minute windows for each of the 90 participants. Colors indicate windows included in the selected 21-day analysis period (dark gray; 322,220 windows in total), valid windows outside this period (light gray; 82,146), and windows excluded by quality filters (red; 5,242). (B) Breakdown of excluded windows: invalid beat count (<150 or >1,000; 3,344), invalid SDNN (<10 ms or >200 ms; 1,857), and missing activity flag (41). Participants are grouped as control (C), meditation (M), and running (R) and ordered by descending total window count. Dashed vertical lines indicate group boundaries.

Multimedia Appendix 3 Normality assessment and data quality summary for questionnaire scores and HRV/HR variables. Shapiro–Wilk test *P* values are reported for the full sample and for each group (C, M, and R). The number of missing observations and the number of IQR-based outliers are also shown.

Multimedia Appendix 4 Kruskal–Wallis tests comparing questionnaire scores and HRV/HR variables among the three groups (C: n=39; M: n=19; R: n=32). The table reports the Kruskal–Wallis test statistic (H), two-sided *P* values, and effect sizes quantified as epsilon squared (ε²).

Multimedia Appendix 5 Pairwise group comparisons for questionnaire scores and overall HRV indices using Wilcoxon rank-sum tests. The table reports the U statistic, two-sided *P* values, Shaffer-corrected *P* values for multiple comparisons, and effect sizes (rank-biserial correlation, r; and common-language effect size). Group sizes were C=39, M=19, and R=32.

Multimedia Appendix 6 Pairwise group comparisons for activity-state RMSSD and heart rate (sleep, rest, walk, and run) using Wilcoxon rank-sum tests. The table reports the U statistic, two-sided *P* values, Shaffer-corrected *P* values for multiple comparisons, and effect sizes (rank-biserial correlation, r; and common-language effect size).

Multimedia Appendix 7 Age-adjusted group comparisons using analysis of covariance (ANCOVA). For each outcome, the table reports the F statistic for the group effect, two-sided *P* values, and effect sizes quantified as partial eta squared (η²). As a sensitivity analysis, *P* values based on HC3 heteroskedasticity-consistent standard errors are also provided. The *P* value for the Group × Age interaction term is reported to assess the homogeneity of regression slopes assumption.

Multimedia Appendix 8 Binomial tests on the proportions of mid-high stress. ESM stress ratings were dichotomized (no-low vs mid-high). For each activity, the proportion of mid-high stress is shown for all participants (All) and for the control (C), meditation (M), and running (R) groups, together with the number of events (k), total observations (n), and estimated proportions (p̂). Two-sided binomial tests assessed differences from the corresponding overall group proportion (Total), with *P* values adjusted using the Holm procedure and effect sizes reported as Cohen's h. Rows labeled All_ref, C_ref, M_ref, or R_ref indicate reference proportions and therefore have no *P* values or effect sizes. The bottom two rows (M vs C and R vs C) compare the meditation and running groups with the control group across all activities, using C_ref as the reference.

Multimedia Appendix 9 Welch’s ANOVA comparing questionnaire scores and HRV/HR variables among the three groups (C: n=39; M: n=19; R: n=32) as a parametric sensitivity analysis. The table reports the Welch’s F statistic with between-groups degrees of freedom fixed at 2, adjusted within-groups degrees of freedom (Adjusted df), two-sided *P* values, and effect sizes quantified as partial eta squared (ηp²).

Multimedia Appendix 10 Games-Howell post hoc tests comparing questionnaire scores and overall heart rate variability (HRV) indices among the three groups (C: control; M: meditation; R: running). The table reports the t statistic, adjusted degrees of freedom (Adjusted df), two-sided *P* values, and effect sizes quantified as Hedges' g.

Multimedia Appendix 11 Games-Howell post hoc tests comparing activity-specific heart rate variability (HRV) and heart rate (HR) indices among the three groups (C: control; M: meditation; R: running). The table reports the t statistic, adjusted degrees of freedom (Adjusted df), two-sided *P* values, and effect sizes quantified as Hedges' g.

Multimedia Appendix 12 Probabilistic graphical representation of the model used for the comparison of RMSSD between stress levels or meditation phases. Circles and rectangles represent random variables and fixed constants, respectively. Shaded squares indicate observed data while a shaded circle indicates a variable of interest. Solid and dashed arrows represent probabilistic and deterministic dependencies, respectively. For definition of variables, see Methods.

Multimedia Appendix 13 Probabilistic graphical representation of the model used for the time-series analysis of RMSSD along meditation practices. Presented as in Multimedia Appendix 12.

Multimedia Appendix 14 Comparative analysis of additional HRV indices and activity-state heart rate. Violin plots illustrate group comparisons of (A) SDNN, (B) meanNN, (C) pNN50, and (D) HR stratified by four physical activity states (sleep, rest, walk, and run; derived from meanNN). Figure presentation and statistical tests were conducted in the same manner as in Figure 2.

Multimedia Appendix 15 Overviews of the ESM data. (A) A bar chart showing the counts of ESM responses across participants. Bar height reflects the total number of responses for each participant. Groups are presented from left to right as control (C), meditation (M), and running (R), with participants within each group ordered by the number of responses in descending order. (B) A stacked bar chart showing the proportions of seven daily activity states for each of the three groups. Bar widths are scaled according to the total number of ESM responses from participants in each group.

Multimedia Appendix 16 Posterior density plots of differences in averaged RMSSD at mid-high stress conditions with reference to no-low stress conditions. In the MCMC sampling process used to generate Figure 4B, posterior distributions were simultaneously estimated for differences in parameters corresponding to averaged RMSSD (αₘ) between the different stress levels. The posterior densities are presented as in Figure 4B. The vertical highlighted dotted line indicates no difference, and negative values on the horizontal axis indicate a reduction in RMSSD under mid-high stress compared to no-low stress.

Multimedia Appendix 17 Overviews of the meditation records. (A) A stacked bar chart showing the number of meditation practices recorded by participants in the meditation groups. Each bar is composed of stacked segments, with each segment representing a single meditation practice from an individual participant. The height of each bar reflects the total count of meditation practices, with participants arranged in descending order along the horizontal axis based on their total count. Within each bar, stacked segments are also ordered in descending duration, with darker shades representing longer meditation practices. (B) A bar chart showing the distribution of meditation practice durations. Practices recorded by all the meditation group members are categorized into 10-minute intervals based on their duration, with the height of each bar representing the total number of practices whose durations fall within each interval. Bar colors correspond to the coding scheme shown in (A).

Multimedia Appendix 18 Posterior density plots of differences in averaged RMSSD at each meditation phase with reference to the "before" meditation phase. In the same manner as in Multimedia Appendix 16, posterior density plots of parameters αₘ were generated from the MCMC sampling process used to generate Figure 5A. A positive value on the horizontal axis indicates an increase in RMSSD under each meditation phase compared to the before meditation phase.

Multimedia Appendix 19 Frequency-domain HRV indices during and after meditation. (A) and (B) Posterior distributions of log-scaled HF and LF are shown in the same manner as Figure 5A. (C) and (D) Baseline-adjusted shifts (differences from the pre-meditation baseline) of log-scaled HF and LF are shown in the same manner as Multimedia Appendix 18.

## Abbreviations

ANCOVA: analysis of covariance
BBI: beat-to-beat interval
ECG: electrocardiography
ESM: experience sampling method
HF: high-frequency
HR: heart rate
HRV: heart rate variability
IPAQ: International Physical Activity Questionnaire
LF: low-frequency
MAAS: Mindful Attention Awareness Scale
MCMC: Markov Chain Monte Carlo
meanNN: mean of normal-to-normal interval
mHealth: mobile health
PPG: photoplethysmography
PSQI: Pittsburgh Sleep Quality Index
PSS: Perceived Stress Scale
RespHRV: respiration-related heart rate variability
RMSSD: root-mean-square of successive differences
SDNN: standard deviation of normal-to-normal interval
WHO-5: World Health Organization Well-being Index

## Data Availability

The datasets generated and analyzed during this study are available from the corresponding author on reasonable request.
