# Supplementary figures and images for: Daily Stress and Heart Rate Variability Among Mindfulness Meditation Practitioners: mHealth Observational Study
Source: J Med Internet Res. 2026 May 29;28:e78244. doi: 10.2196/78244 (PMC13263660; doi:10.2196/78244)

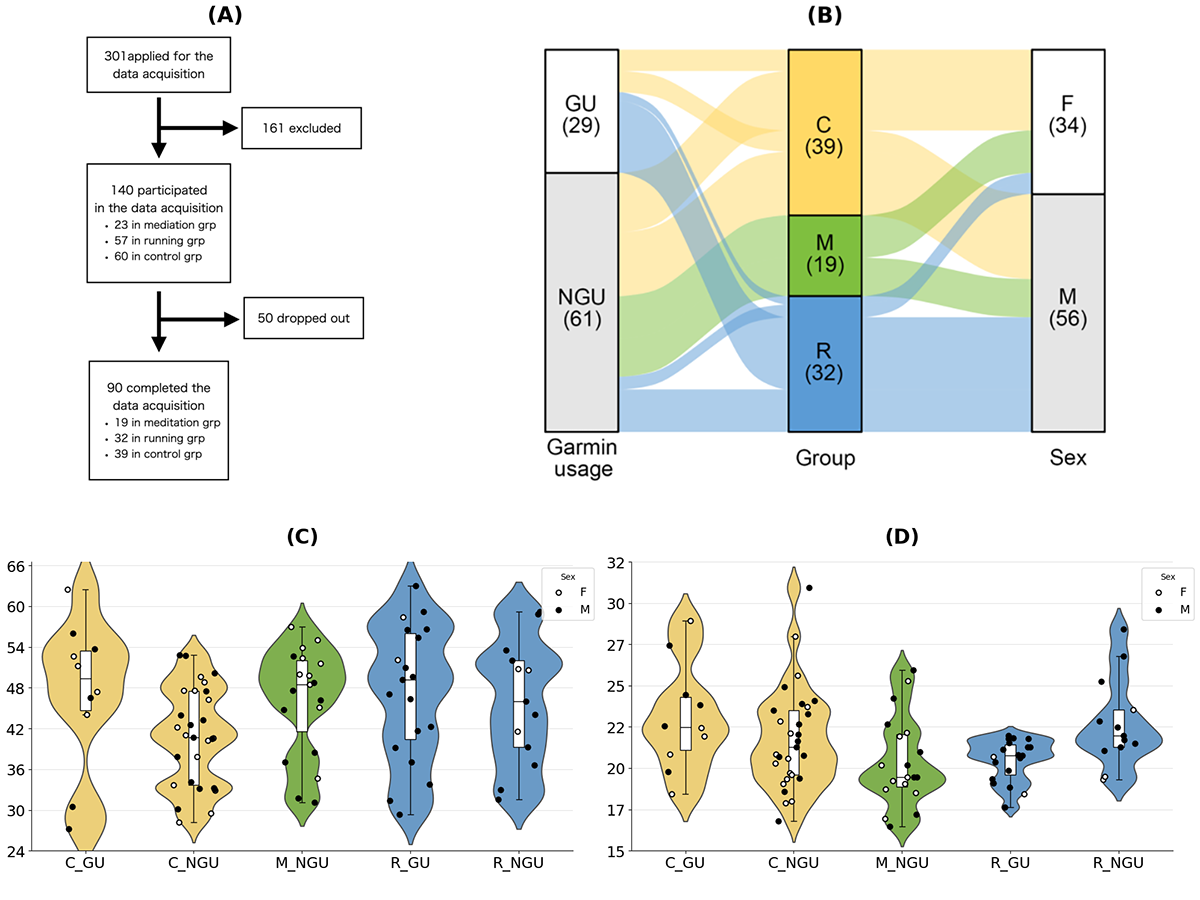

Supplement: Multimedia Appendix 1 [file jmir_v28i1e78244_app1.png]

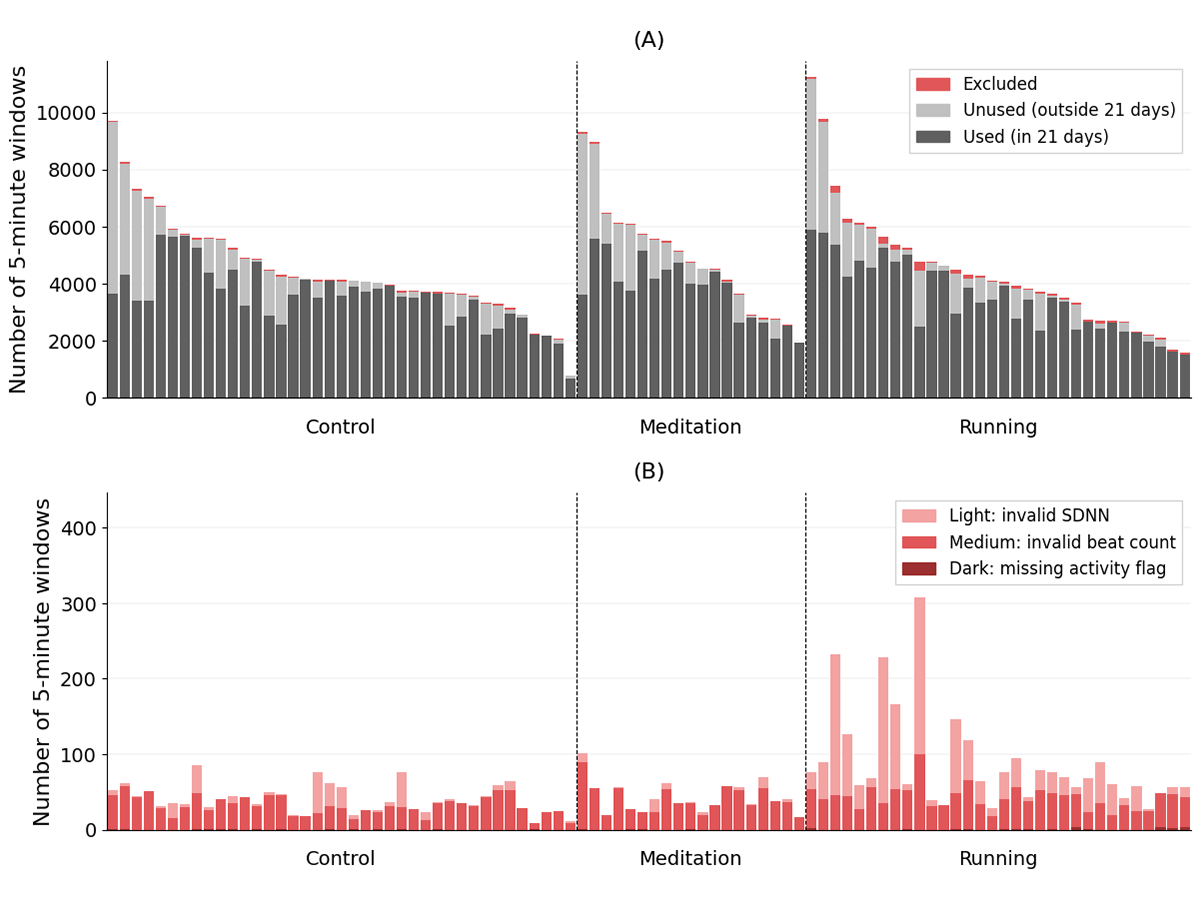

Supplement: Multimedia Appendix 2 [file jmir_v28i1e78244_app2.png]

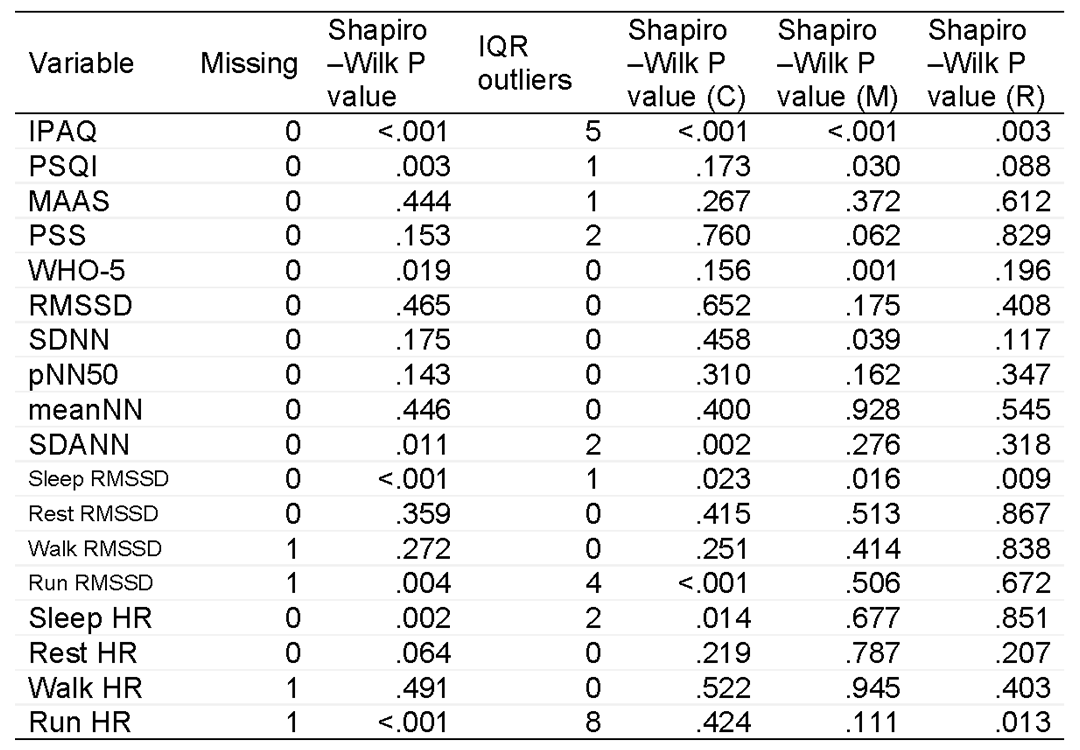

Supplement: Multimedia Appendix 3 [file jmir_v28i1e78244_app3.png]

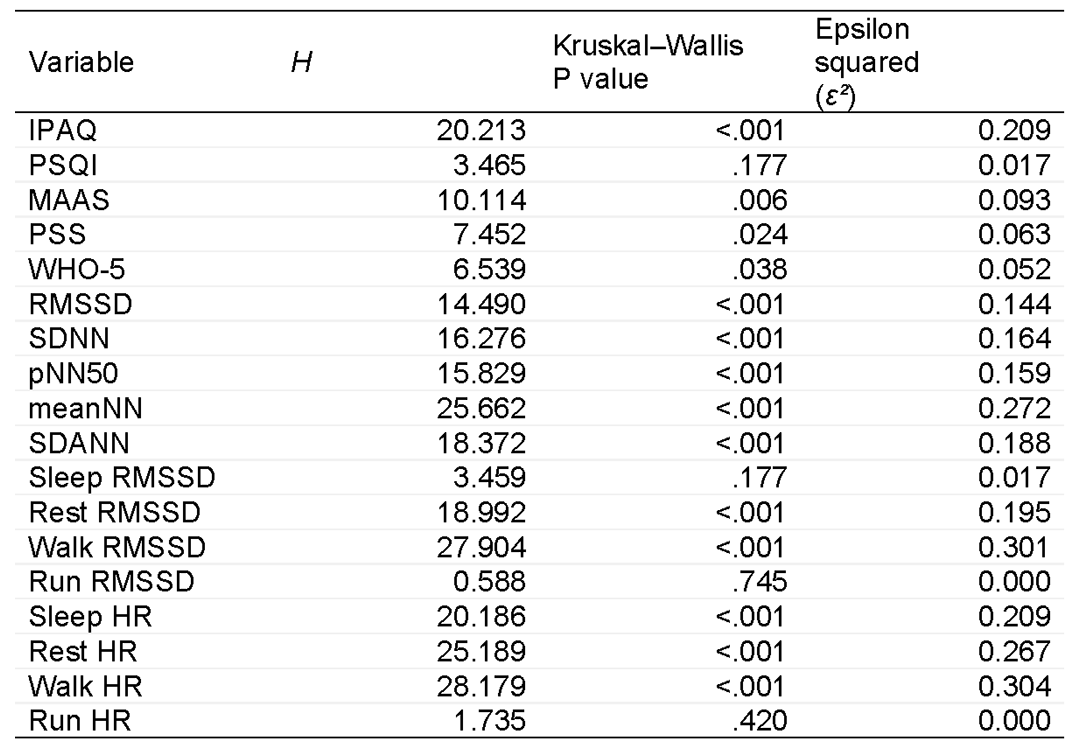

Supplement: Multimedia Appendix 4 [file jmir_v28i1e78244_app4.png]

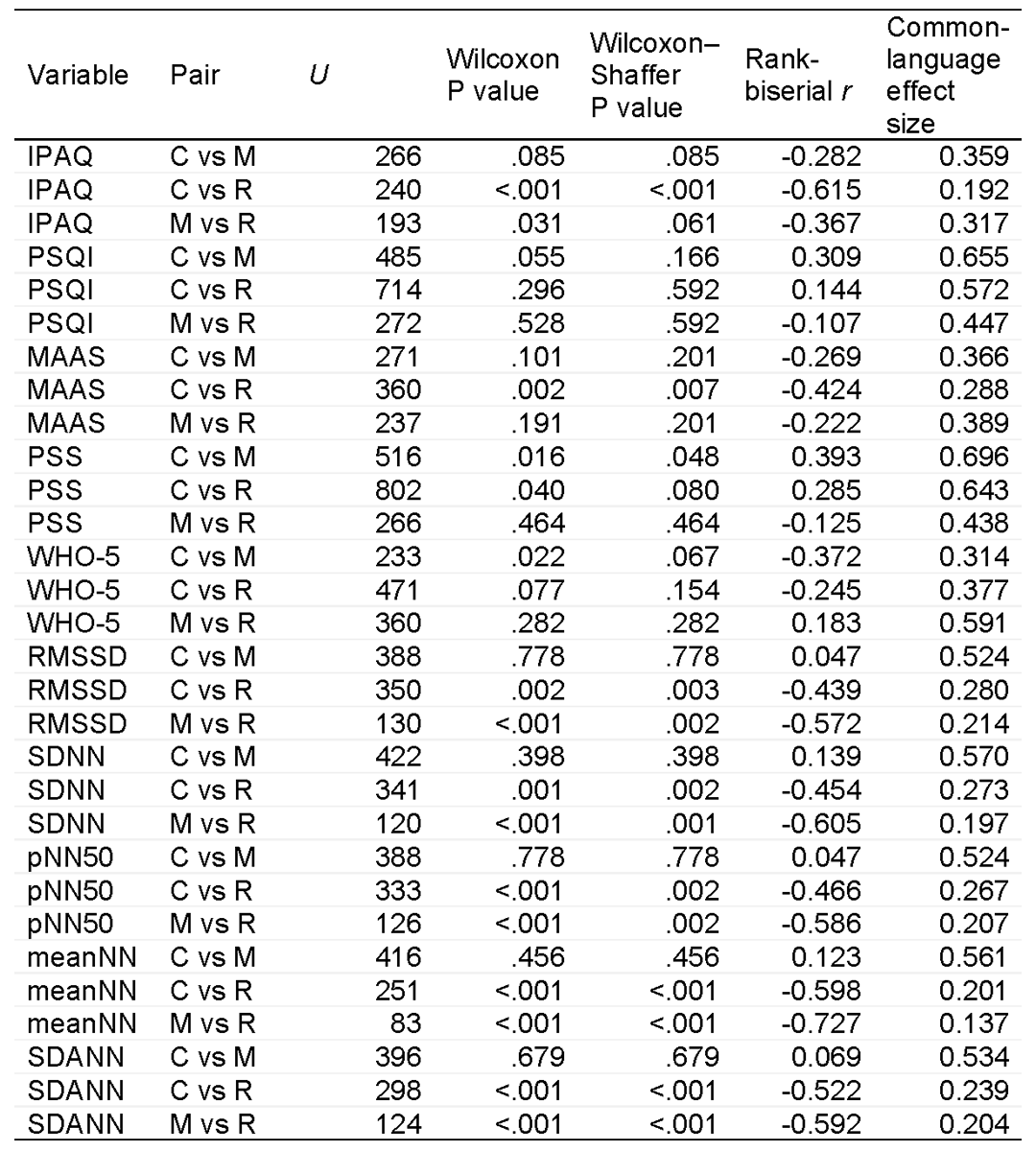

Supplement: Multimedia Appendix 5 [file jmir_v28i1e78244_app5.png]

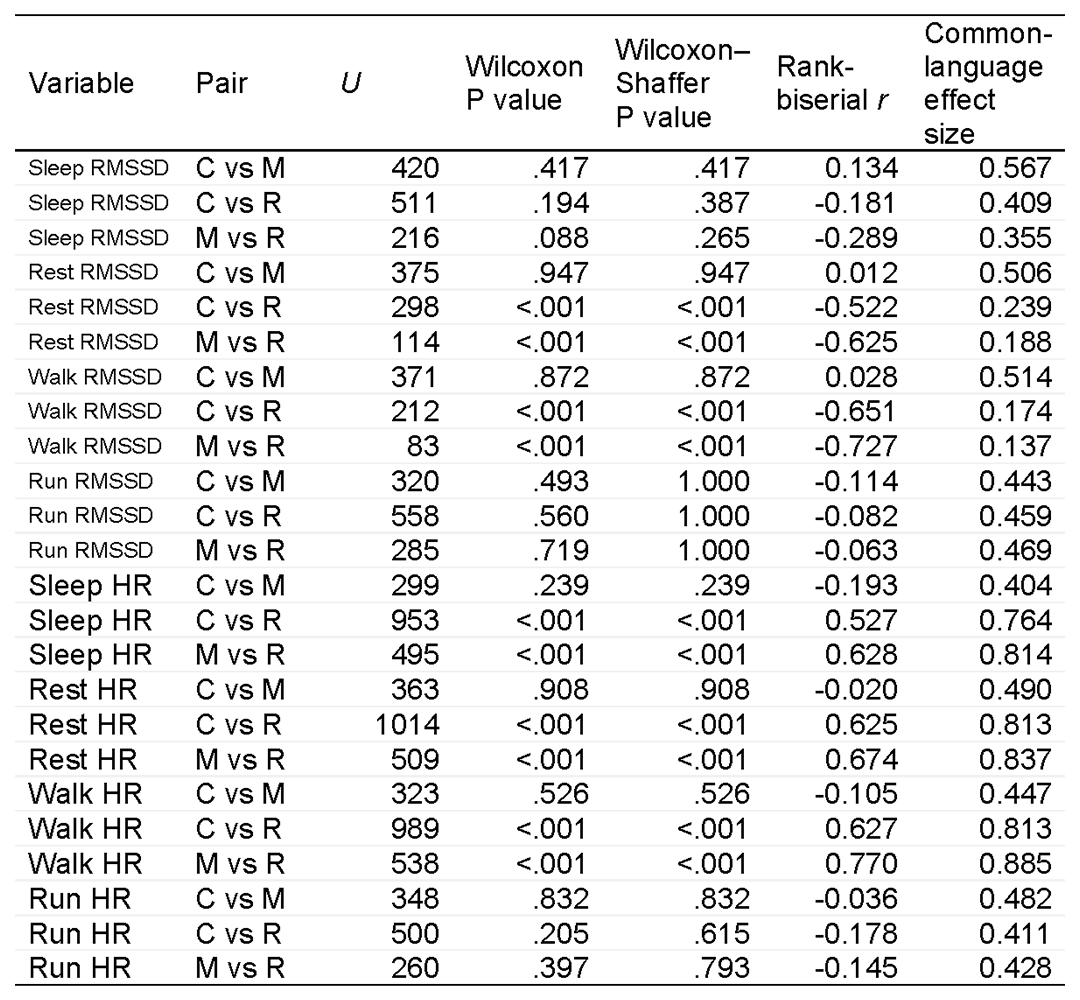

Supplement: Multimedia Appendix 6 [file jmir_v28i1e78244_app6.png]

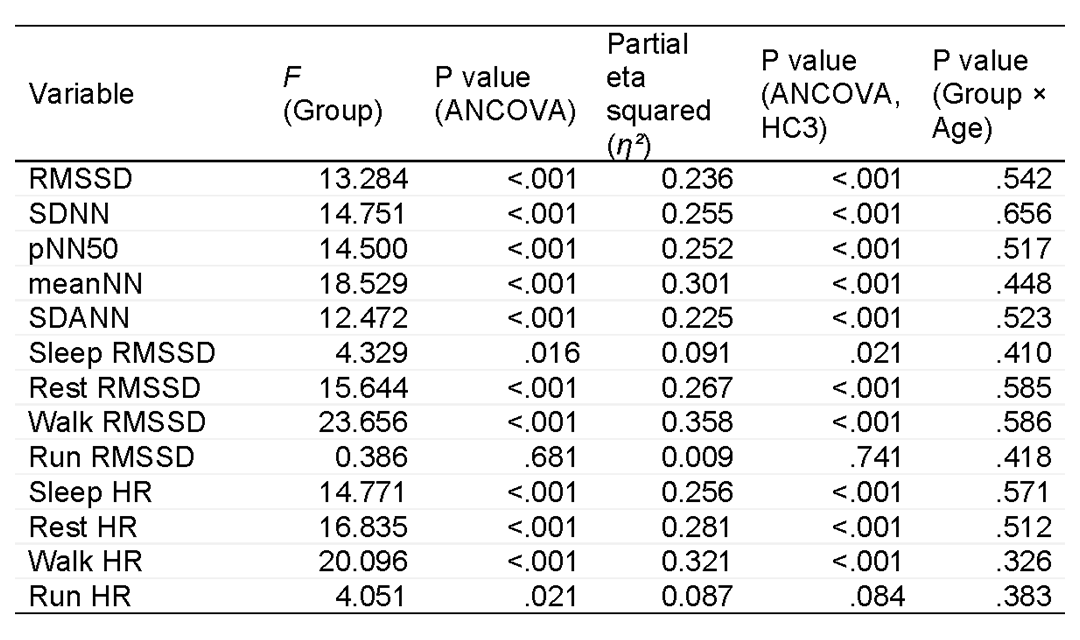

Supplement: Multimedia Appendix 7 [file jmir_v28i1e78244_app7.png]

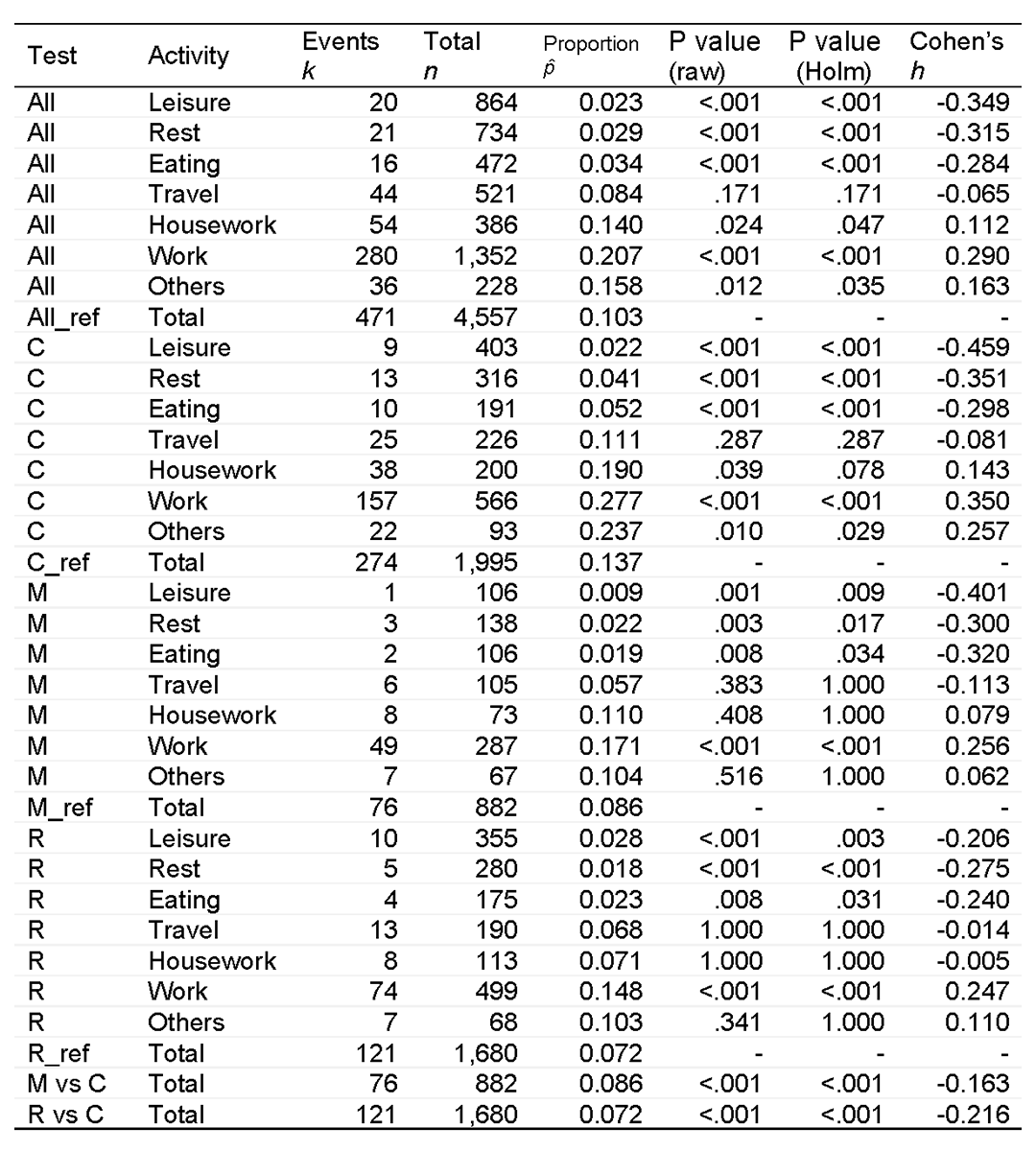

Supplement: Multimedia Appendix 8 [file jmir_v28i1e78244_app8.png]

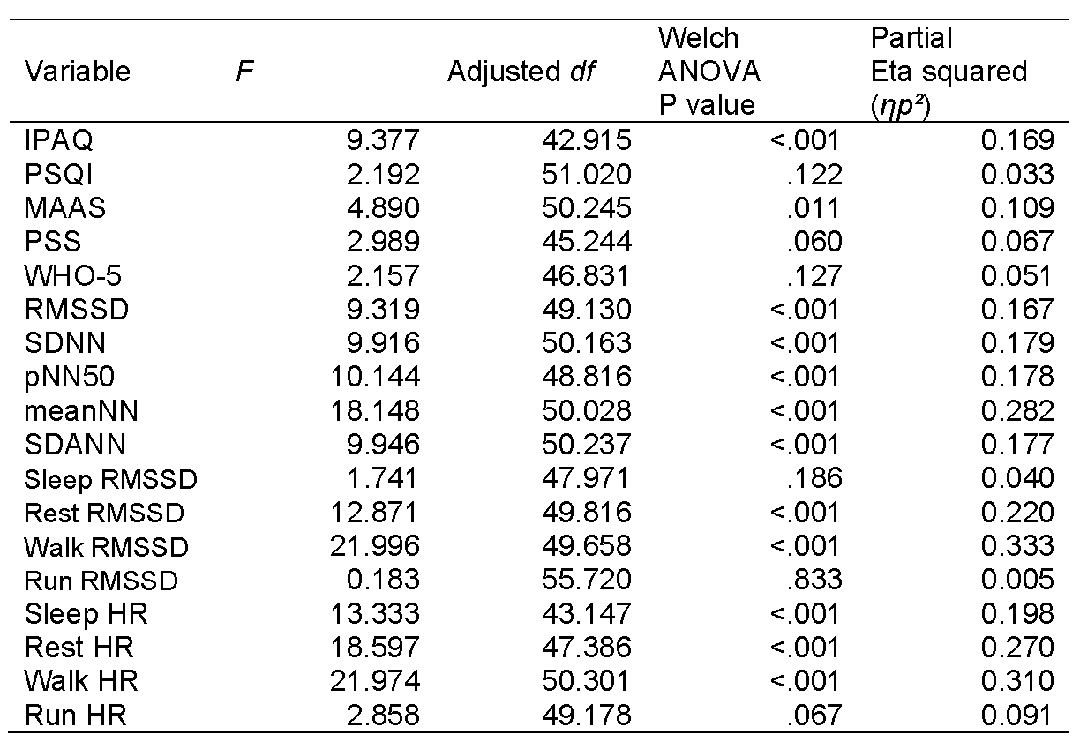

Supplement: Multimedia Appendix 9 [file jmir_v28i1e78244_app9.png]

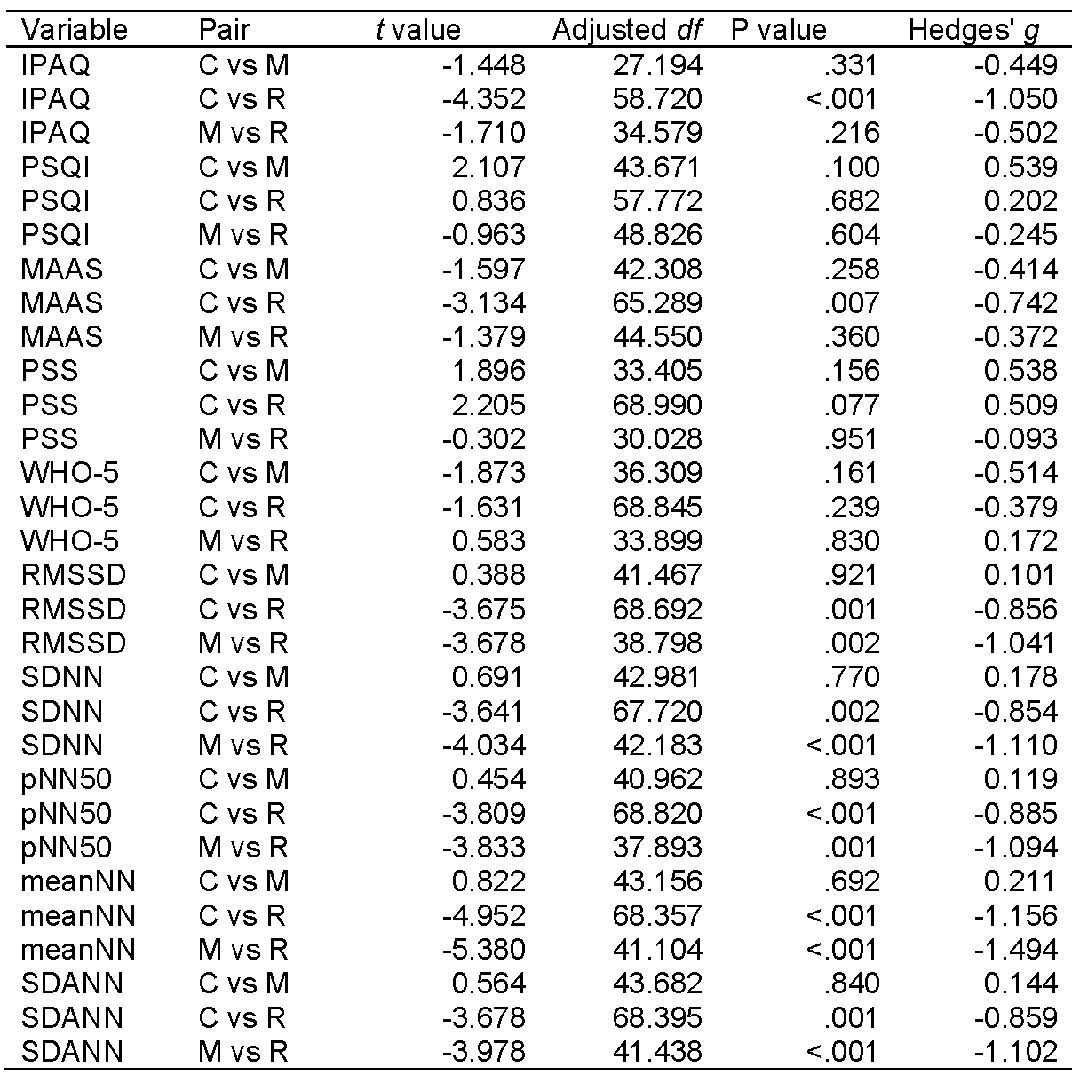

Supplement: Multimedia Appendix 10 [file jmir_v28i1e78244_app10.png]

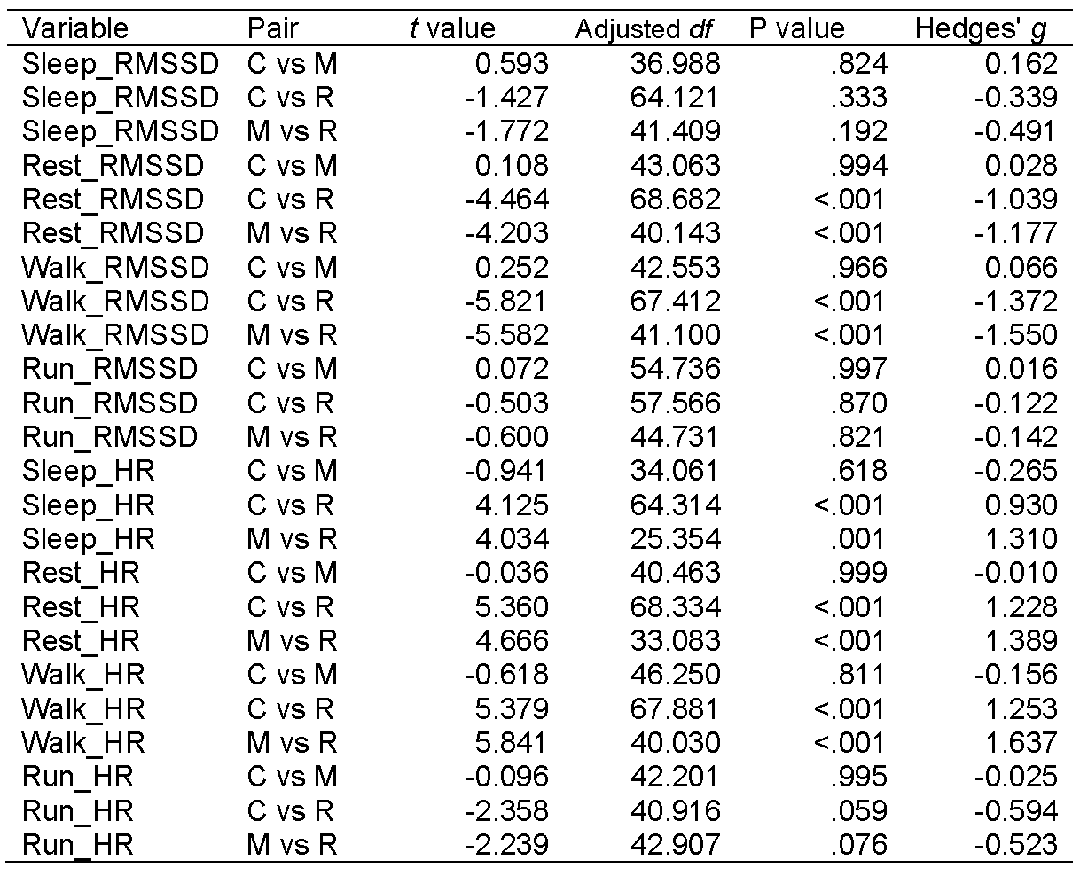

Supplement: Multimedia Appendix 11 [file jmir_v28i1e78244_app11.png]

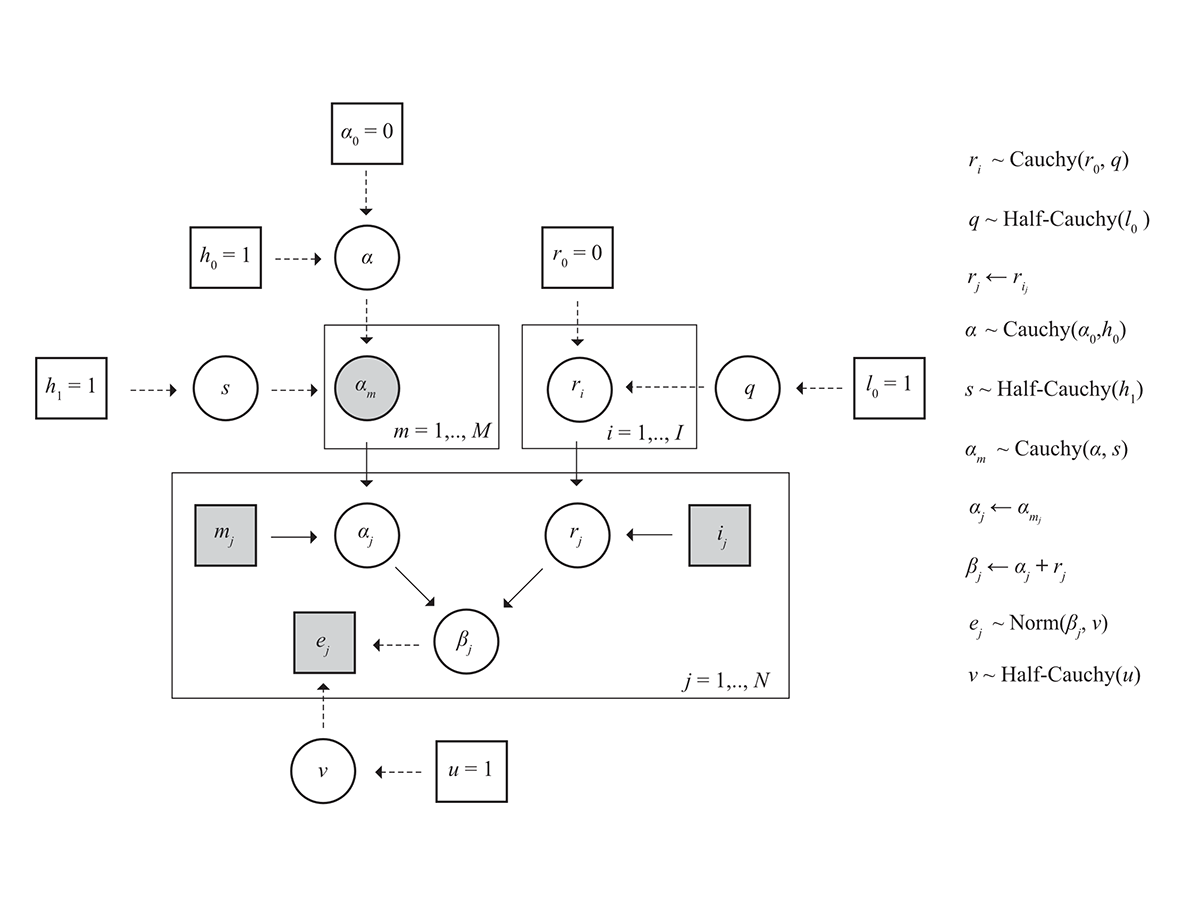

Supplement: Multimedia Appendix 12 [file jmir_v28i1e78244_app12.png]

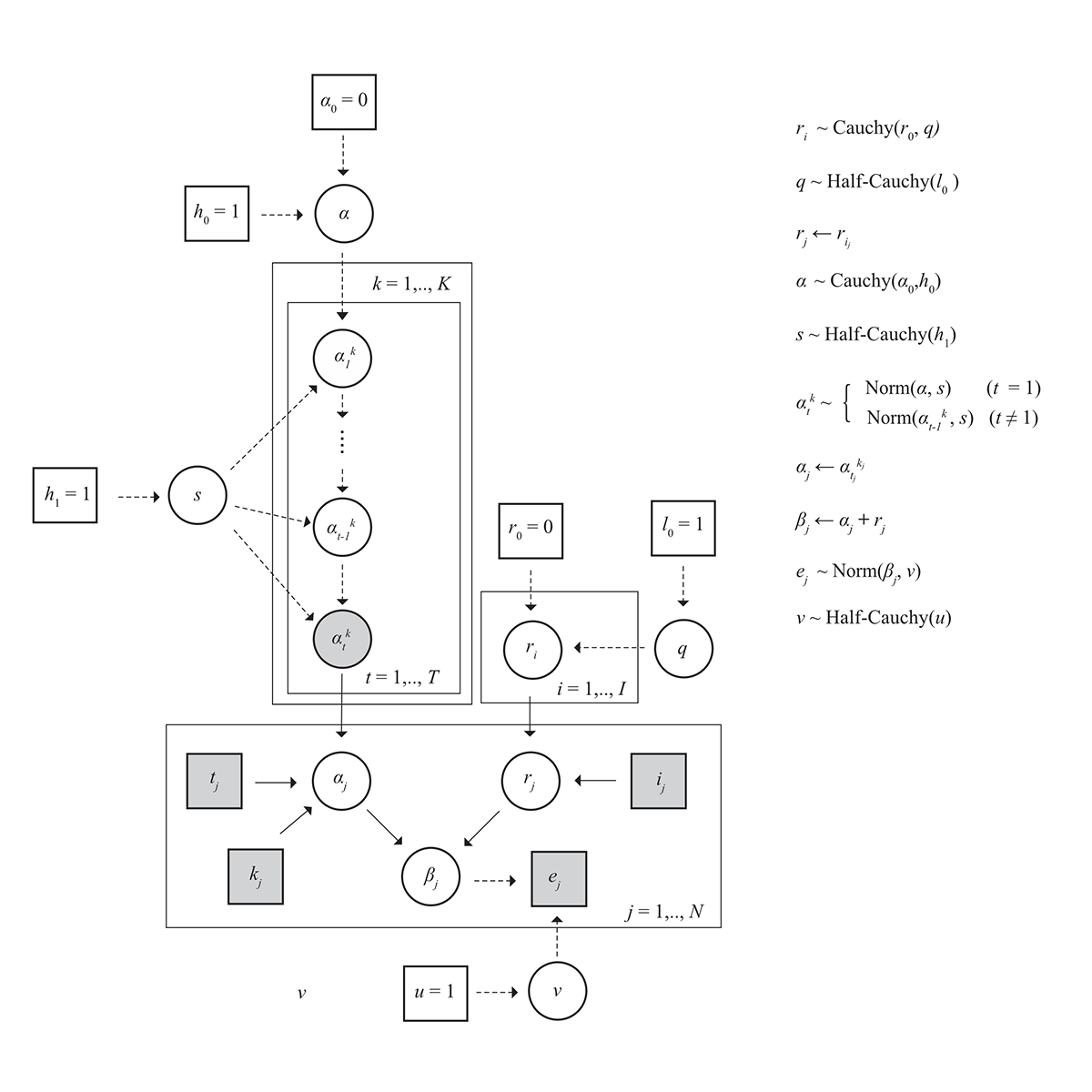

Supplement: Multimedia Appendix 13 [file jmir_v28i1e78244_app13.png]

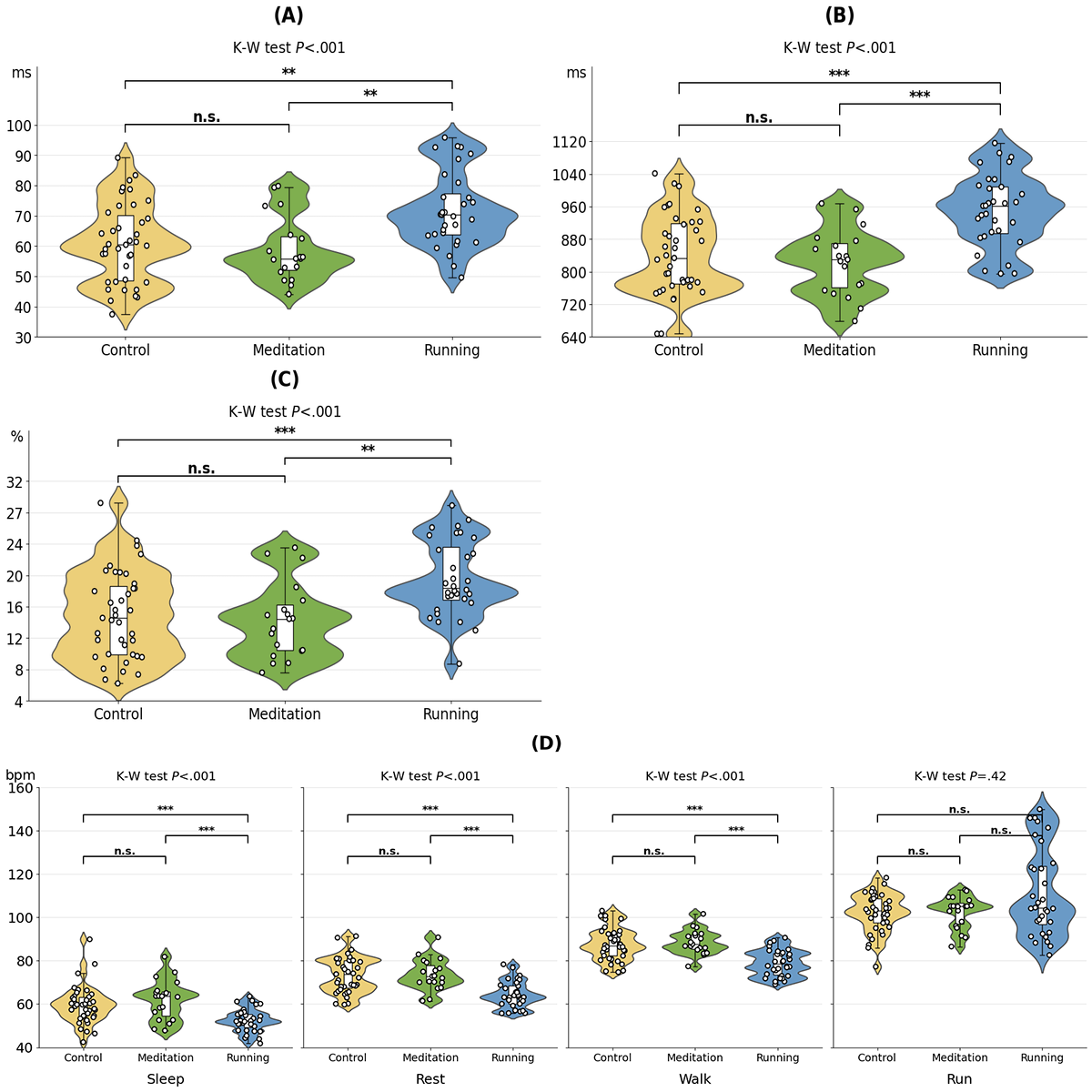

Supplement: Multimedia Appendix 14 [file jmir_v28i1e78244_app14.png]

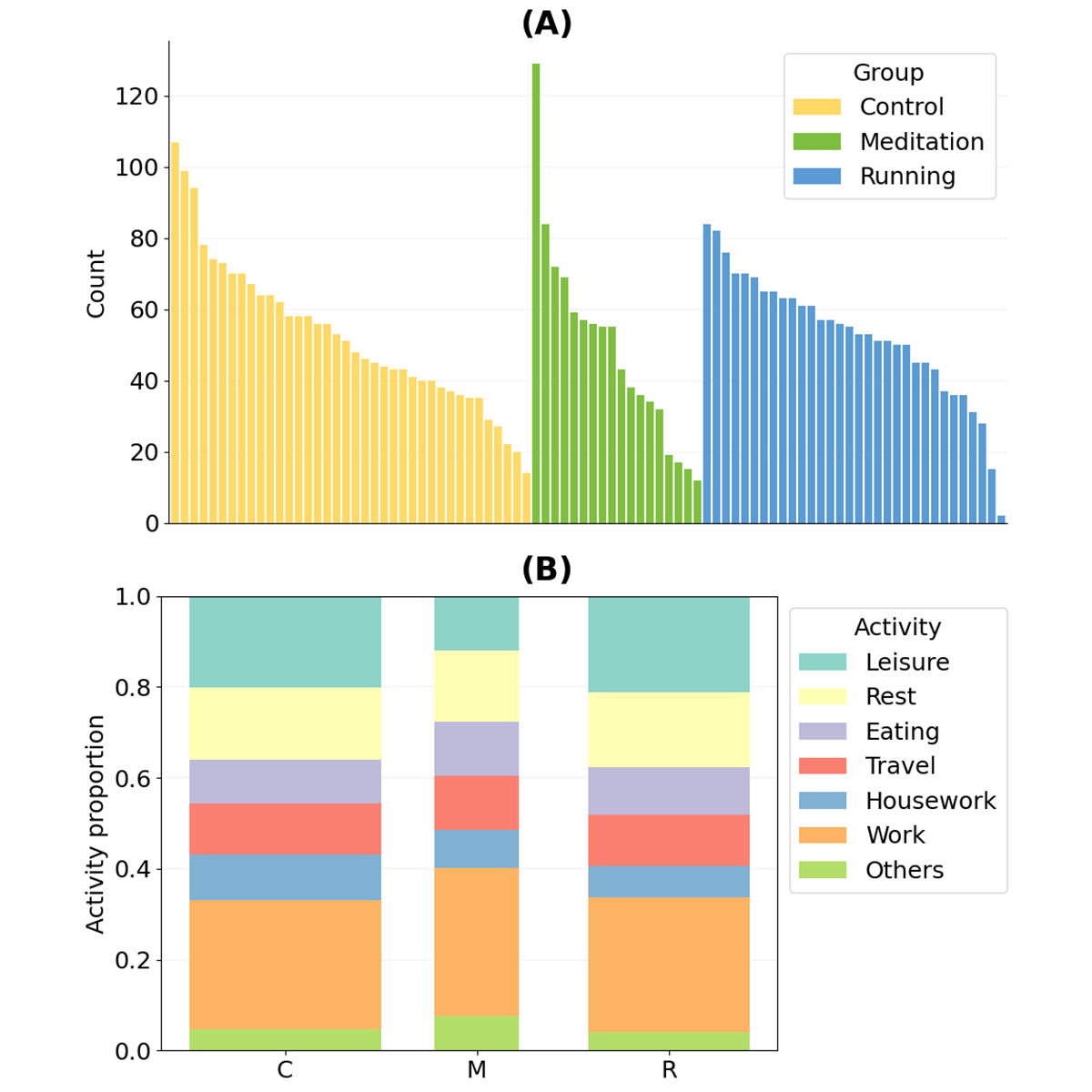

Supplement: Multimedia Appendix 15 [file jmir_v28i1e78244_app15.png]

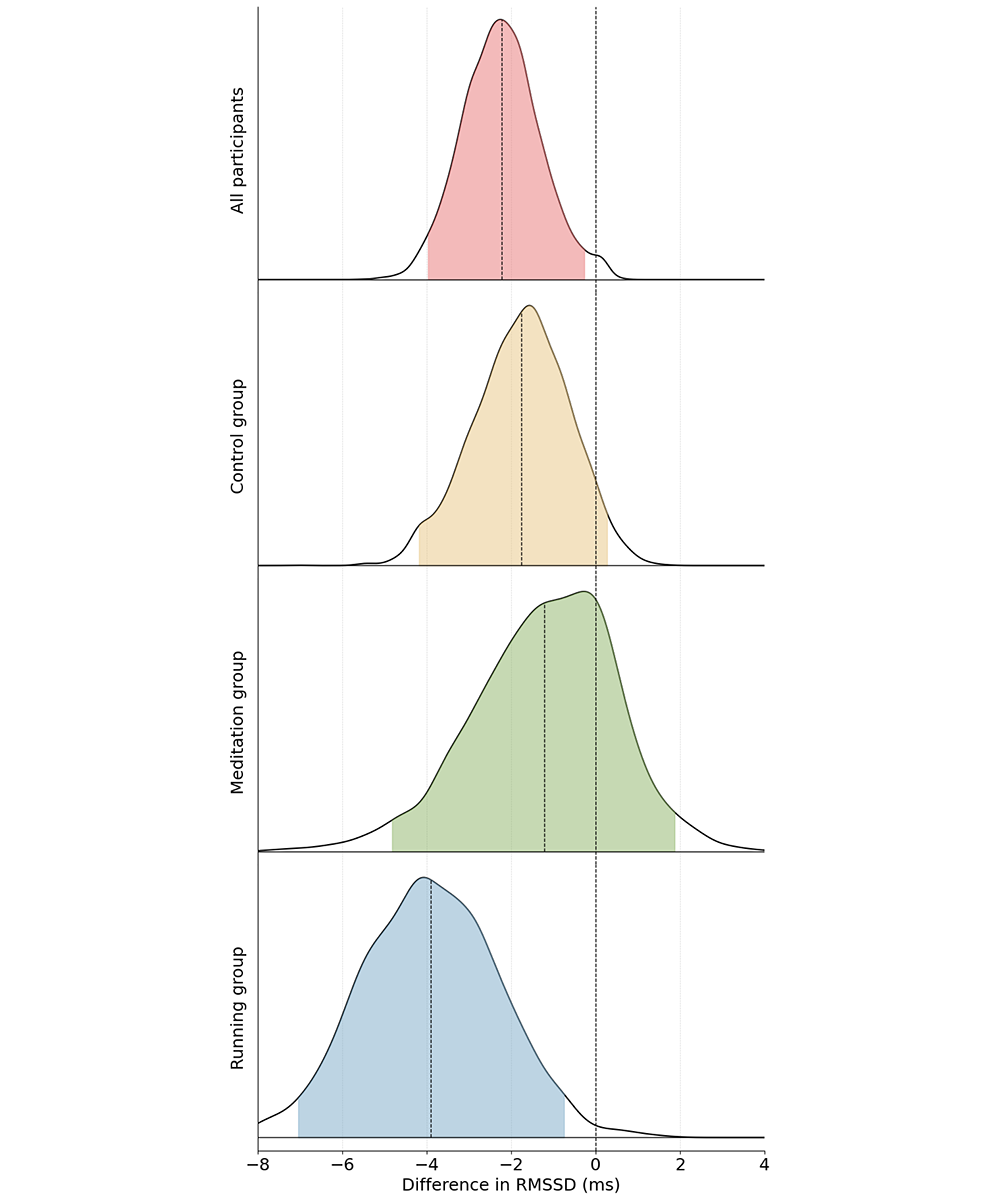

Supplement: Multimedia Appendix 16 [file jmir_v28i1e78244_app16.png]

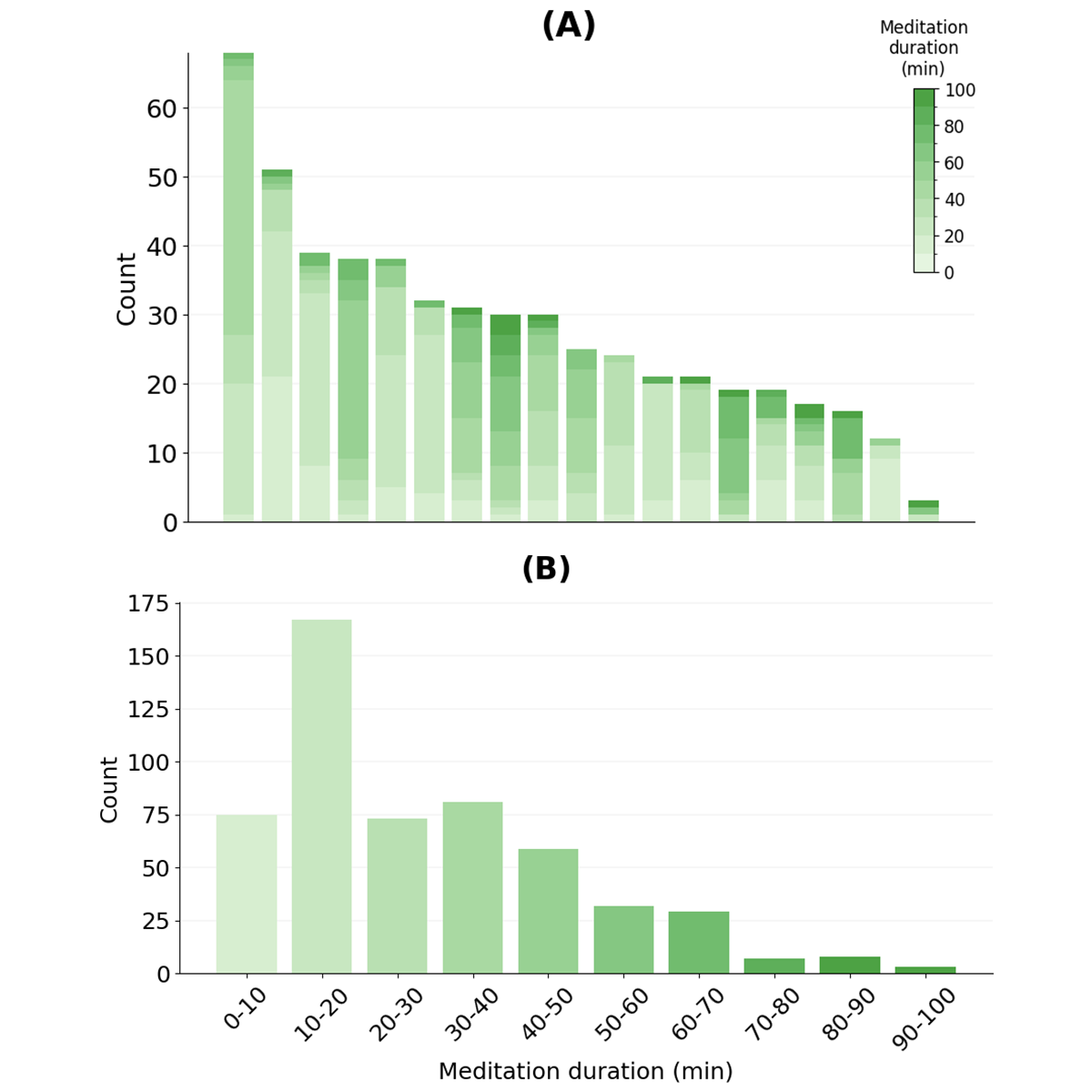

Supplement: Multimedia Appendix 17 [file jmir_v28i1e78244_app17.png]

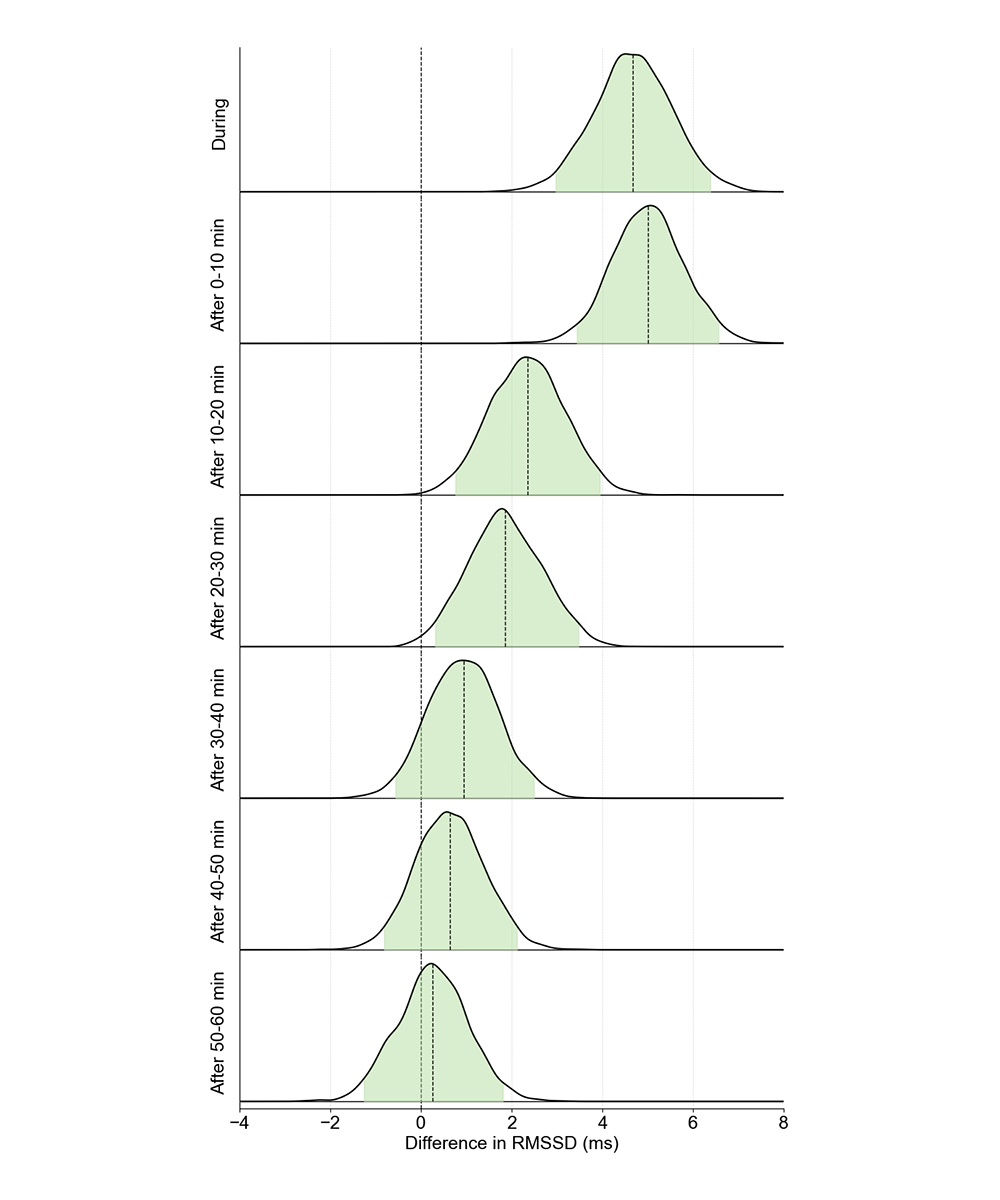

Supplement: Multimedia Appendix 18 [file jmir_v28i1e78244_app18.png]

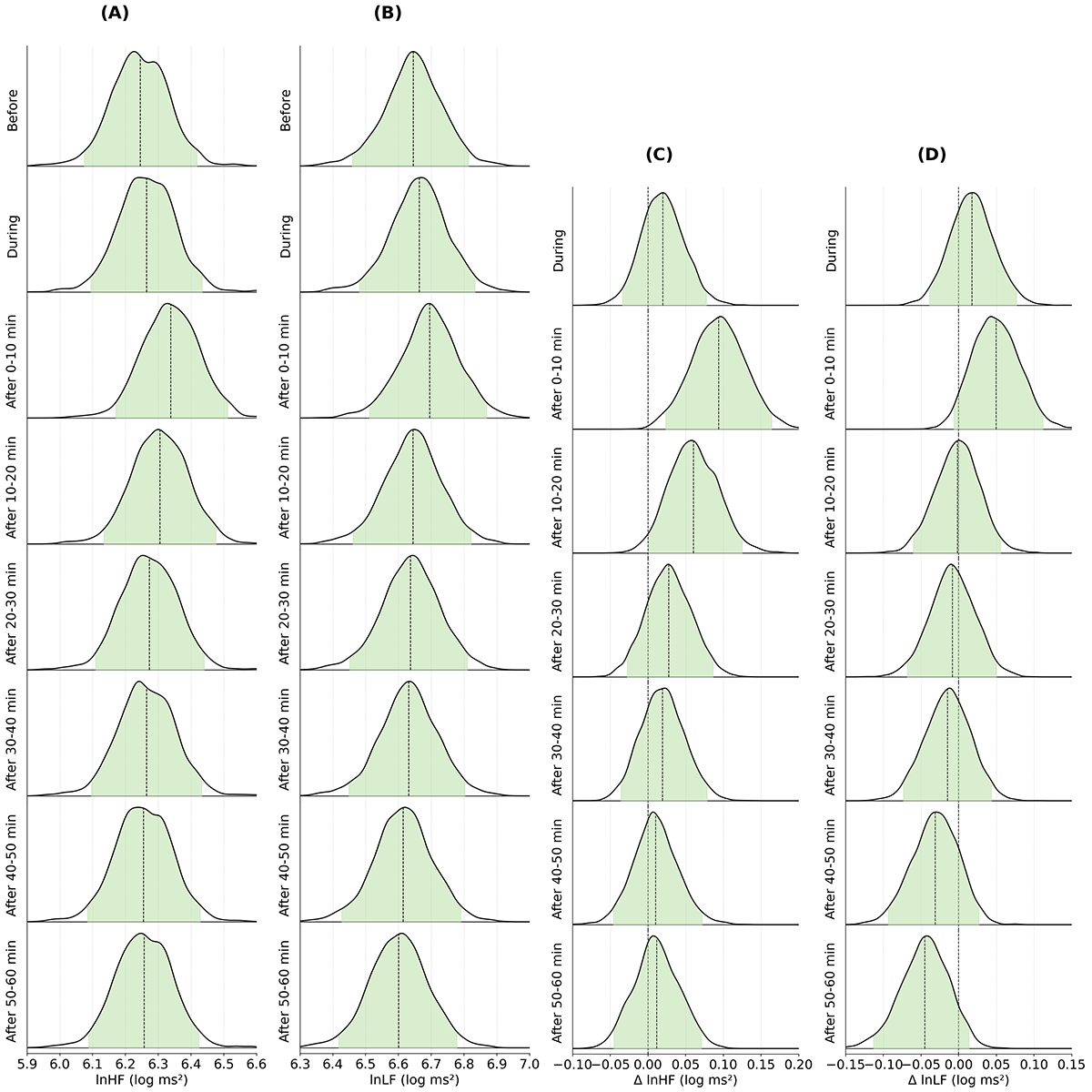

Supplement: Multimedia Appendix 19 [file jmir_v28i1e78244_app19.png]
